# A Developmental Transcriptome Map for Allotetraploid *Arachis hypogaea*

**DOI:** 10.3389/fpls.2016.01446

**Published:** 2016-09-30

**Authors:** Josh Clevenger, Ye Chu, Brian Scheffler, Peggy Ozias-Akins

**Affiliations:** ^1^Institute of Plant Breeding, Genetics, and Genomics, University of GeorgiaTifton, GA, USA; ^2^United States Department of Agriculture - Agricultural Research Service, Genomics and Bioinformatics Research UnitStoneville, MS, USA

**Keywords:** transcriptomics, *Arachis hypogaea*, developmental co-expression networks, homeolog expression bias, alternative splicing, non-coding RNA, PRJNA291488

## Abstract

The advent of the genome sequences of *Arachis duranensis* and *Arachis ipaensis* has ushered in a new era for peanut genomics. With the goal of producing a gene atlas for cultivated peanut (*Arachis hypogaea*), 22 different tissue types and ontogenies that represent the full development of peanut were sequenced, including a complete reproductive series from flower to peg elongation and peg tip immersion in the soil to fully mature seed. Using a genome-guided assembly pipeline, a homeolog-specific transcriptome assembly for *Arachis hypogaea* was assembled and its accuracy was validated. The assembly was used to annotate 21 developmental co-expression networks as tools for gene discovery. Using a set of 8816 putative homeologous gene pairs, homeolog expression bias was documented, and although bias was mostly balanced, there were striking differences in expression bias in a tissue-specific context. Over 9000 alterative splicing events and over 6000 non-coding RNAs were further identified and profiled in a developmental context. Together, this work represents a major new resource for cultivated peanut and will be integrated into peanutbase.org as an available resource for all peanut researchers.

## Introduction

Cultivated peanut (*Arachis hypogaea*) is an important grain legume worldwide, ranking second in production among all grain legumes and fifth among oilseeds (fao.faostat.org, 2013). An allopolyploid (AABB type genome; 2*n* = 4*x* = 40), modern peanut evolved from the hybridization and subsequent chromosome doubling of two diploid species *A. duranensis* (A) and *A. ipaensis* (B) (Kochert et al., 1996; Seijo et al., 2007). *A. duranensis* and *A. ipaensis* diverged only 2.5 – 3.5 million years ago, which is more recent than other allopolyploid progenitor genomes such as cotton (6.7 mya) or wheat (6.9 mya) (Senchina et al., 2003; Devos et al., 2005; Moretzsohn et al., 2013; Bertioli et al., 2016). Due to the recent divergence, the subgenomes of peanut are very similar to one another (Bertioli et al., 2016). The close relationship of the subgenomes leads to many challenges with transcriptome assembly. Homeologous gene sequences between subgenomes become collapsed into consensus transcripts. This difficulty has led to incomplete transcriptome resources for *A. hypogaea*.

Recently, the genomes of *A. duranensis* and *A. ipaensis* have been sequenced (Bertioli et al., 2016). The *A. ipaensis* accession sequenced, K30076, was found to be a probable direct descendant of the population that contributed to the polyploidization of *A. hypogaea* (Bertioli et al., 2016). Comparisons of *A. hypogaea* cv Tifrunner B genome and *A. ipaensis* reveal similarity of 99.96%. Similarity between the A genome and *A. duranensis* is 98.66% (Bertioli et al., 2016). The high similarity to the tetraploid subgenomes make these genome sequences excellent resources for guiding *A. hypogaea* homeolog-specific transcriptome assemblies.

Two strategies for homeolog-specific assembly have been proposed in wheat (Krasileva et al., 2013; Ranwez et al., 2013). Based on the idea that homeologs will be differentially regulated, Ranwez et al. (2013) propose splitting contigs with apparent high heterozygosity into homeologous sequences by using the most represented base pairs in one and the least represented in the alternative (Ranwez et al., 2013). The downside of this strategy is that gene pairs that are not differentially expressed will be incorrectly split. Krasileva et al. (2013) propose a strategy that utilizes remapping of reads to assembled transcripts and phasing reads to assemble homeolog-specific sequences. This strategy is promising, but SNPs can be incorrectly phased with short reads due to loss of information between sequences. The high similarity between the sequenced genome of *A. ipaensis* and *A. hypogaea*'s B genome and *A. duranensis* and the A genome of peanut provides an opportunity to use a genome-guided assembly strategy for homeolog-specific assembly. This strategy has been employed and demonstrated to be highly accurate previously (Bertioli et al., 2016).

In the absence of a tetraploid reference genome sequence for cultivated peanut, the landscape of expressed sequences is an important resource for genetics, genomics, and molecular breeding. A unique and fascinating aspect of peanut development is geocarpy, the development of the seed underground after pollination of the flower above ground (Moctezuma, 2003). Peanut is the only major crop that exhibits geocarpic development. Maturation in peanut cultivation is important for yield characteristics including high oleic acid ratio and grade. To date there is no mutation for determinate reproduction, as peanuts set pegs up until harvest. Knowledge of the genes and gene networks that control flowering and geocarpic development in peanut will allow targeting of genes toward producing determinant peanuts, which would be of great value to growers worldwide. This is just a single example of the impact a comprehensive gene atlas will have on peanut molecular genetics, genomics, and breeding.

With the goal of establishing an expression atlas for *A. hypogaea*, deep RNA sequencing of 22 tissue types and ontogenies of the reference genome cultivar (cv Tifrunner) was conducted. Over 3 billion paired-end Illumina reads were produced, and a homeolog-specific assembly of transcribed sequences was generated using a genome-guided assembly strategy. Twenty-one gene networks regulating the full scope of peanut development were annotated. Using our homeolog-specific assembly, we profiled global subgenome expression bias, identified over 9000 alternative splicing events (AS) and profiled their usage in a tissue-specific manner, and identified over 6000 long non-coding (lnc) RNAs. Altogether this work is a benchmark for expression dynamics in *A. hypogaea* providing a homeolog-specific transcriptome assembly, global view of alternative splicing, and identification of lncRNAs for cultivated peanut. The resources described herein will be publicly available in peanutbase.org.

## Materials and methods

### Plant material and RNA extraction

**Figure 1:**
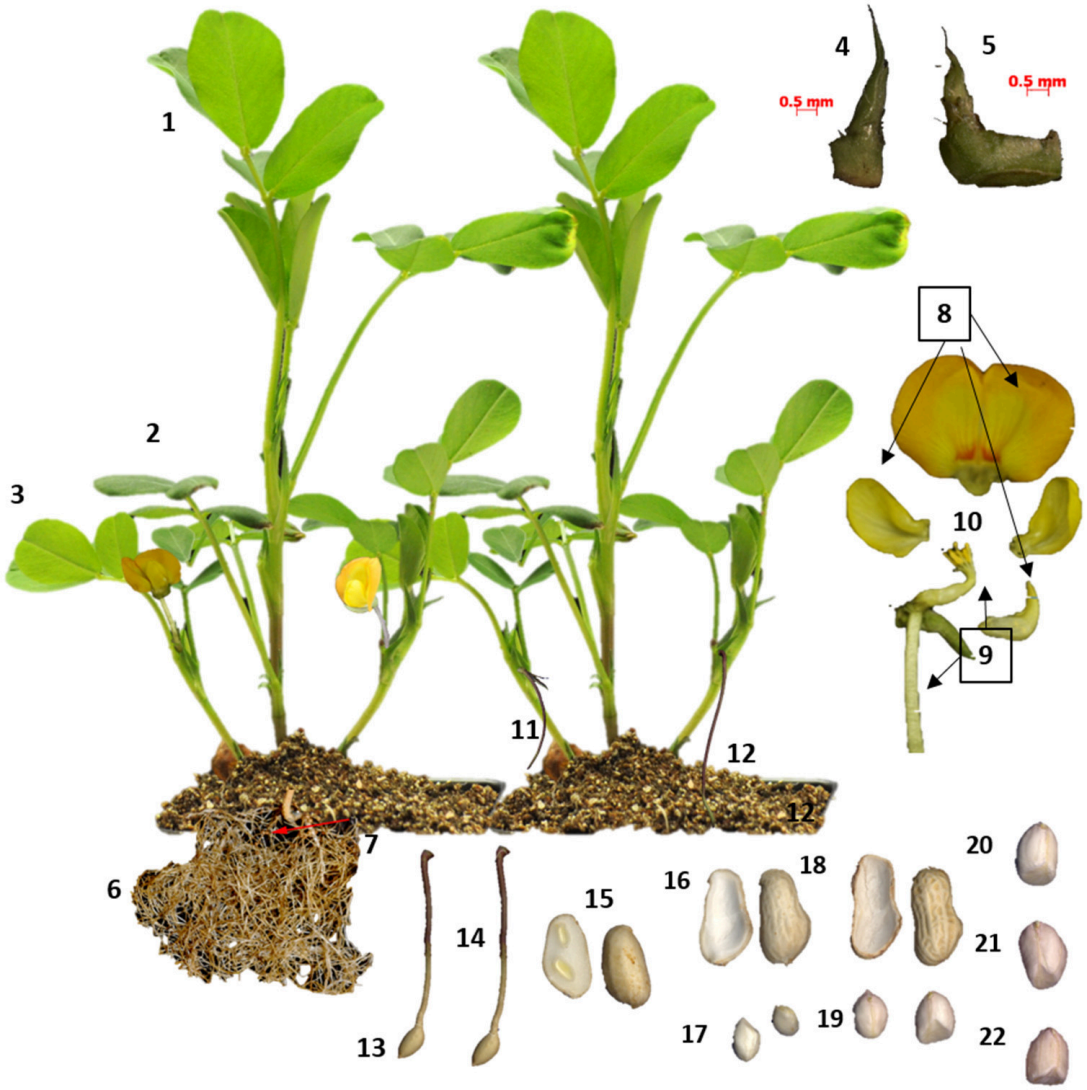
**Tissues sampled from cv Tifrunner**. (1) seedling leaf 10d post emergence (2) main stem leaf (3) lateral (n+1) leaf (4) vegetative shoot tip from main stem (5) reproductive shoot tip from first lateral (n+1) (6) 10d roots (7) 25d nodules (8) perianth (9) gynoecium (10) androecium (11) aerial gynophore tip (12) subterranean gynophore tip (24 h) (13) Pattee 1 pod (14) Pattee 1 stalk (15) Pattee 3 pod (16) Pattee 5 pericarp (17) Pattee 5 seed (18) Pattee 6 pericarp (19) Pattee 6 seed (20) Pattee 7 seed (21) Pattee 8 seed (22) Pattee 10 seed.

RNA was extracted from cultivar Tifrunner (Holbrook and Culbreath, 2007), the genotype that is being sequenced currently for the peanut genome project (http://www.peanutbioscience.com/peanutgenomeinitiative.html). Growth stage description for collection of leaf and shoot tissue followed Boote's classification (Boote, 1982). Developmental stages of peanut pods (fruit) during initiation and maturation were determined according to Pattee et al. (1974) in which peanut kernel maturity was classified into 15 categories. Pod collection was performed for up to stage 10 out of 15 (Figure 1). Tissue description is listed in Table S1. Images of tissue collected for RNAseq analysis is illustrated in (Figure 1).

Peanut plants were grown in the greenhouse (24–30°C) from March to August of year 2012. Peanut seeds were treated with Vitavax PC (Bayer CropScience, Research Triangle Park, NC) and germinated in 50% Promix potting mix (Premier Horticulture Inc., Quakertown, PA) and 50% steam-sterilized sandy soil from the Coastal Plain Experiment Station, University of Georgia Tifton Campus (Tifton, Georgia) fertilized with Osmocote 14-14-14. Rhizobium (*Bradyrhizobium spp*; Peanut Special; EMD Crop Bioscience) was inoculated in the soil prior to planting. All harvests were performed at 14:00 h except for flower samples which were collected at 8:30 h. Three biological replicates of each tissue type were collected from three different plants. Tissue samples were flash frozen in liquid nitrogen and stored at −80°C. Peanut flowers were dissected into three parts, i.e., gynoecium (including stigma, style and ovary), androecium (staminal tube, filaments and anthers) and perianth (wings, banner, hypanthium, keel and lower lip of the calyx) prior to freezing. Peanut pods were harvested and washed thoroughly with sterilized deionized water. Peanut pods from Pattee stages 1 and 3 to 4 were collected intact. Pericarp and kernel samples were separated for pods at Pattee stages 5 to 6. Kernel samples only were collected for pods at Pattee stages 8 and 10.

Root and nodule tissues were harvested from plants grown in a growth chamber (16 h/8 h of day/night cycle; 22°C; 50 to 60% relative humidity). Vitavax-treated seeds were planted in vermiculite at a rate of one seed/pot. Rhizobium (Peanut Special; EMD Crop Bioscience) was applied in the soil prior to planting. Peanut seedlings were watered with a modified B&D solution (Broughton and Dilworth, 1971) without nitrogen once or twice a week prepared as follows: 1 L of 18 mOhm water, 1 ml of solution A (1 M CaCl_2_), 1 ml of solution B (0.5 M KH_2_PO_4_), 0.5 ml of solution C (20 mM Fe EDTA), and 1 ml of solution D (0.25 M MgSO_4_, 0.25 M K_2_SO_4_, 1 mM MnSO_4_, 2 mM H_3_BO_4_, 0.5 mM ZnSO_4_, 2 mM CuSO_4_, 0.1 mM CoSO_4_, and 0.1 mM Na_2_MoO_4_). The pH of the solution was adjusted to 5.8 before use. Roots were harvested 10 days post emergence and nodules were harvested 25 days post emergence.

Peanut tissue samples were ground in liquid nitrogen using a mortar and pestle. Total RNA was extracted with the RNeasy plant mini kit (Qiagen, Valencia, CA). Total RNA was treated with DNaseI (Life Technologies, Grand Island, NY) and concentrated by RNeasy mini-elute cleanup kit (Qiagen). RNA quality was checked by gel electrophoresis and analysis on an Agilent 2100 Bioanalyzer performed at the Georgia Genomics Facility (Athens, GA). RNA samples with RIN number greater than 8 were included for library construction.

### Library construction and sequencing

Libraries were constructed as in Bertioli et al. (2016). Briefly, TruSeq RNA Sample Preparation v2 kits were used for library construction and paired-end 2 × 100 bp sequencing was carried out on an Illumina HiSeq 2500 instrument with a total of 209 cycles of TruSeq Rapid SBS Kit v1 (Illumina) chemistry.

### Genome-guided assembly

Assembly was conducted using a genome-guided approach in the assembly pipeline from Trinity v. 2.0.6 r2013-08-14 with slight modification. First an *in silico* amphidiploid genome was created by simply disregarding scaffolds and concatenating the *A. duranensis* genome assembly with the *A. ipaensis* genome assembly naming each simply “A” and “B.” Then normalized reads (normalized to maximum coverage of 50x using Trinity Normalization Haas et al., 2013) were mapped to this genome using GSNAP (Wu and Watanabe, 2005) with the following parameters, “-N 1 -w 10,000 -n 20 -t 6 -nofails,” where –N turns on novel splicing, -w 10,000 decreases default local splicing distance parameter (default 20,000), -n 20 allows mapping to 20 different paths (default 100), -t 6 determines number of threads to use, and—nofails outputs only aligned reads. Once the reads were mapped, the SAM file was run through the genome-guided pipeline. Briefly, loci of reads were extracted into separate directories where they were assembled on a locus by locus basis using the following parameters “–seqType fa –JM 2G –CPU 4 –genome_guided –run_as_paired.” Then the assembled transcripts were concatenated together into one assembly. Information of whether loci were derived from *A. duranensis* or *A. ipaensis* was retained so transcripts were annotated as either “A”- or “B”-derived during concatenation of the final assembly.

### Expression-based filtering of final assembly

Total reads were mapped to the transcript assembly from 58 libraries (22 distinct tissue types and developmental stages including vegetative and seed) using Bowtie allowing 2 mismatches in a 25 bp seed. Fragments per kilobase per million reads mapped (FPKM) were estimated using RSEM (Li and Dewey, 2011) for each library. When reads map to multiple transcripts, RSEM fractionates the read count among the transcripts so read counts are not integers. Transcripts were filtered out that had less than 1 FPKM for all 58 libraries using filter_fasta_by_rsem_values.pl from the Trinity package, and were deemed lacking in minimum read coverage evidence to be supported.

### Redundancy filtering

Expression-based filtered transcripts were tested for redundancy using a custom script in order to retain locus information from the assembly in the transcript names. Filtering was done using an intra-subgenome self-blast. Transcripts with 90% or greater coverage and 100% identity were filtered out, leaving the longer transcript.

### Repetitive sequence filtering

Transcripts were aligned to a set of annotated repetitive sequences from *A. duranensis* and *A. ipaensis* supplied by Dr. David Bertioli using GMAP (Wu and Watanabe, 2005) with the following parameters; “-n 4” where –n controls the number of paths. The classes of repetitive sequence were as follows; LINEs, Ty-3-gypsy LTR, TY1-copia LTR, and MITEs (*A. hypogaea*).

### Assembly accuracy using diagnostic sequence

*A. duranensis* and *A. ipaensis* pseudomolecules were fragmented into 100 bp fragments using the command “perl -ne ‘BEGIN{$/ = “>”}{s/(.^*^)//;$ n = $1;s/\n//g;$i = 0;s/(.{1100})/printf(“>%s_%05d\n%s\n,”$n,++$i,$1);/ge;}.” These fragments were then mapped to their opposite genome using Bowtie. The SAM files were filtered for those fragments that mapped uniquely and completely (no clipping) with only one mismatch to the opposite genome. These fragment sequences were collected as diagnostic sequences. To test the accuracy of the assembled transcripts, diagnostic sequences were mapped to the transcript assembly using GSNAP with the following paramters: “-n 1 -m 0 -A sam –nofails.” Fragments diagnostic for “A” and mapping completely with no mismatch to “A”-derived transcripts were counted as correct and those mapping to “B”-derived transcripts were counted as ambiguous. This was also done for “B” diagnostic transcripts.

### Redundancy reduction using the evidential gene pipeline

Further redundancy reduction was carried out using the Evidential Gene pipeline (http://arthropods.eugenes.org/genes2/about/EvidentialGene_trassembly_pipe.html; Nakasugi et al., 2014). The subsequent assembly is referred to as GG-RED.

### Homology-based annotation of assembled transcripts

Annotation was carried out using the Trinotate pipeline (http://trinotate.github.io/). Briefly, Transdecoder (Haas et al., 2013) was used to predict protein coding ORFs. BLASTx was used to query transcripts and Transdecoder predicted protein coding regions against the Uniprot database. HMMER (Finn et al., 2011) and signalP (Petersen et al., 2011) were run to identify Pfam (Finn et al., 2013) domains and to predict signal peptides, respectively. Finally TMHMM (Krogh et al., 2001) was run to identify transmembrane domains. The final annotation included gene ontologies (GO) from BLAST and domain results.

### Construction of gene developmental networks

Expression was calculated using RSEM (Li and Dewey, 2011). RSEM uses maximum likelihood modeling to estimate the expected expression taking into account reads that map to multiple transcripts. FPKM was used as normalized expression. To find developmental gene networks, the 22 tissue types and ontogenies were clustered into three groups. Seedling leaf, main stem leaf, lateral stem leaf, vegetative shoot tip, roots, and nodules were grouped as the vegetative stages. Reproductive shoot tip, perianth, gynoecium, androecium, aerial peg tip, subterranean peg tip, Pattee 1 pod, Pattee 1 stalk, and Pattee 3 pod were grouped as the reproductive stages. The final group, seed development, was made up of Pattee 5 seed, Pattee 5 pericarp, Pattee 6 seed, Pattee 6 pericarp, Pattee 7 seed, Pattee 8 seed and Pattee 10 seed. The average of all replicates was taken and further normalized as a z-score across all samples in each group.

Developmental gene networks were identified using Self-Organizing Maps (SOM) in R with the Kohonen package. Vegetative SOMs were identified with a 3 × 2 hexagonal SOM grid and five SOMs were identified as physiologically relevant. These SOMs are referred to as Vegetative Network (VN) I – V. Reproductive SOMs were identified with a 5 × 4 hexagonal SOM grid and nine SOMs were identified as physiologically relevant. These SOMs are referred to as Reproductive Network (RN) I – IX. Seed development SOMs were identified with a 4 × 3 hexagonal SOM grid and seven SOMs were identified as physiologically relevant. These SOMs are referred to as Seed Development Network (SN) I – VII.

GO term enrichment for each SOM was carried out by hypergeometric enrichment test with the R function, phyper(), and p values were adjusted for multiple testing with a Bejamini-Hochberg correction.

### Homeolog identification and subgenome expression bias

A set of 8816 putative homeolog pairs were identified using reciprocal BLAST (Table S2) with the GG-RED transcript set. Filtering of matches was done using awk scripting for pairs that had matches greater than 80% base pair identity and greater than 80% length coverage. Additional criteria were best reciprocal match of the predicted amino acid open reading frame (ORF) and predicted coding sequence (CDS). Pairs that met these criteria were determined to be putative homeolog pairs.

Subgenome expression bias was evaluated with the rounded estimates of raw counts for each transcript in the 8816 pairs for all samples. Differential expression between pairs was calculated with DESeq2 (Love et al., 2014) and adjusted for multiple testing with a Benjamini-Hochberg correction.

### Ka/Ks estimations

Predicted ORFs from Transdecoder were aligned using ClustalW. Ka/Ks and Ks for homeologous pairs was calculated with PAML (yn00) (Yang, 2007). Wilcox Signed-Rank Test in R (wilcox.test()) was used to calculated p values between biased and unbiased homeologous pairs.

### Alternative splicing

Reads were mapped to a concatenated *A. ipaensis* and *A. duranensis* genome (Bertioli et al., 2016; peanutbase.org; pseudomolecules v1.0) using Tophat2 v. 2.1.0 (Kim et al., 2013). Finesplice (Gatto et al., 2014) was then used to identify all splice junctions present in the data. Any splice junctions that were not supported by at least 10 reads in a single sample were filtered out. Splice junctions from all 58 libraries were then subjected to custom scripts to identify three different types of alternative splicing event: exon skipping, alternative 5′ donor, and alternative 3′ acceptor (Table S3). Alternative 5′ donor events were found when junction start sites were duplicated with unique end sites. Alternative 3′ acceptor events were found when junction end sites were duplicated with unique start sites. Exon skipping events were found when duplicated start sites shared an end site that is also duplicated, but with different start sites.

NAGNAG acceptor events were found by extracting all alternative 3′ acceptor events that had proximal and distal junction sites within 3 base pairs. The sequence 3 base pairs 3′ to the proximal site to the distal site were extracted and searched for the NAGNAG motif.

### Long non-coding RNAs

Transcripts with no predicted open reading frame using Transdecoder and no hit in the Uniprot database were subjected to further analysis. First transcripts with average FPKM lower than 2 were filtered out. Possible ORFs were predicted again using the Coding Potential Calculator (CPC) (Kong et al., 2007) and transcripts with predicted ORFs longer than 80 amino acids were filtered out. Finally, coding potential was calculated again using Coding Potential Assessment Tool (CPAT) (Wang et al., 2013) and 298 transcripts were estimated to have coding potential and eliminated. The final set of 6314 transcripts was further investigated.

Filtered transcripts were queried against Mirbase 21 using BLAST (Kozomara and Griffiths-Jones, 2013). Transcripts retained had hits of greater than 80% identity by nucleotide and greater than 90% coverage of the stem loop (Table S4). These transcripts were determined to be putative pri-miRNA transcripts.

Final putative lncRNAs were mapped to a concatenated genome consisting of the *A. ipaensis* and *A. duranensis* reference pseudomolecules (Bertioli et al., 2016) using gmap v. 2013-09-11 (Wu and Watanabe, 2005). Coordinates were extracted and Bedtools (Quinlan and Hall, 2010) was used to identify overlaps with annotated genes using the *A. ipaensis* and *A. duranensis* gene annotations (Bertioli et al., 2016). Intergenic lncRNAs were determined to not overlap an annotated gene feature and to be 200 base pairs away from any annotated gene (Table S5). Intragenic lncRNAs were described as exonic if they overlapped a predicted exon (Table S6). Intragenic lncRNAs were described as intronic if they overlapped a predicted mRNA, but did not overlap any predicted exon (Table S7).

### Figure construction

All figures were made using R 3.1.1 and the package ggplot2.

### Data availability

All fastq sequences are deposited at the National Center for Biotechnology Information (http://www.ncbi.nlm.nih.gov/) under BioProject PRJNA291488. All raw sequences are deposited as BioSamples SAMN03944933 - SAMN03944990. The reference transcripts are deposited as TSA contigs GDKN01000001 - GDKN01102303.

## Results

### Genome-guided assembly

Genome-Guided (GG) assembly of the transcriptome greatly increased the resolution of homeolog-specific assembly (Bertioli et al., 2016). A total of 351,265 transcripts were assembled initially, of which 47.16% were A-derived and 52.84% were B-derived. Filtering by a minimum expression of 1 FPKM in at least one tissue type/ontogeny across all replicates left 196,734 transcripts. After a conservative redundancy reduction and filtering of known peanut repetitive elements (Bertioli et al., 2016), the final GG assembly consisted of 183,062 transcripts.

One additional assembly strategy and one alternative redundancy reduction pipeline were tested for comparison with the GG assembly (Table 1). For alternative assembly, all normalized reads were first parsed into “A” and “B” specific read sets by mapping to a combined *A. duranensis* and *A. ipaensis* genome. These read sets were assembled separately *de novo* (DNOVO) with Trinity and filtered as described above for the genome-guided assembly. The final *de novo* assembly consisted of 163,004 transcripts. Alternative redundancy reduction was achieved using the EvidentialGene pipeline (http://arthropods.eugenes.org/genes2/about/EvidentialGene_trassembly_pipe.html; Nakasugi et al., 2014). This pipeline is homology-based and was developed to select the best set of transcripts from many assemblies. Using this pipeline on the “A”- and “B”-derived transcripts from the GG assembly separately produced a best set of 80,326 transcripts (Table 1) which is in line with the predicted gene numbers for *A. duranensis* and *A. ipaensis* (Bertioli et al., 2016). This reduced set of transcripts was used for expression analyses and gene network analysis to control for possible misassembled differentially spliced isoforms and over-assembly of large gene families. This assembly is referred to as GG-RED.

**Table 1:** **Assembly stats of genome-guided Trinity assembly, genome-guided assembly filtered with Evigene, and read parsed ***de novo*** assembly**.

	**Genome-guided Trinity**	**Genome-guided Trinity—Evigene**	**SubGenome read parsed *de novo***
Transcripts	183,062	80,326	163,044
N50 (bp)	1829	1805	1800
Average (bp)	1186	1303	1200
Total assembled (bp)	217,208,968	104,737,129	195,793,148
Homeolog-specific mapping^a^	65.87 ± 0.80%	57.24 ± 0.62%	55.80 ± 0.42%
Diploid proteins recovered^b^	35,944	28,312	24,103

a *Average reads mapped back to assembly allowing 0 mismatches for all libraries*.

b *Amount of transcripts that cover >80% length with >90% identity of diploid A. Ipaensis and A. duranensis protein models*.

Previously, different assembly methods were evaluated for accuracy of homeolog-specific assembly (Bertioli et al., 2016). Here we describe a subset of analyses showing that our assembly is highly accurate in terms of resolution of homeologous gene copies. The GG assembly was able to capture an average of 65.87% of reads mapped with no mismatches showing a high degree of homeolog resolution (Table 1). The principle is that in a situation where two homeologous copies are collapsed into a consensus sequence, where a base is polymorphic between them, a consensus base is chosen. This leads to unmapped reads from the alternative sequence if no mismatches are allowed. A more accurate assembly will result in a higher number of mapped reads without mismatches. The GG assembly captured on average 10% more reads than the DNOVO assembly, further showing that Genome-Guided assembly produces superior transcriptome assemblies even when using progenitor genome sequence. Even GG-RED, with almost 100 Mb less sequence assembled, captured on average 2% more reads than DNOVO (Table 1). Further, even with transcript number in line with the predicted gene number, GG-RED captured 8% fewer reads than GG, showing that GG is the most complete assembly. However, 8% of total reads is disproportionately small considering GG contains 100,000 more transcripts and an additional 117 Mb assembled sequence (Table 1).

The assemblies also were evaluated with a set of 396,349 100 bp sequences that were diagnostic for the “A” genome and 437,496 100 bp sequences that were diagnostic for the “B” genome. These diagnostic sequences were obtained by fragmenting the *A. duranensis* and *A. ipaensis* genome sequence into 100 bp fragments. These fragments were then mapped to the opposite genome sequence and filtered for those fragments that mapped only one time completely with only 1 mismatch. These fragments were then gathered as diagnostic sequences for the “A” and “B” genomes. Of these fragments, 56,095 “A” diagnostic sequences mapped to expressed sequences and 70,741 “B” diagnostic sequences mapped to expressed sequences. After mapping these sequences to GG contigs with no mismatches allowed, 94.32% of “B” diagnostic reads mapped correctly to a “B”- derived transcript while 99.62% of “A” diagnostic reads mapped correctly to an “A”-derived transcript showing high accuracy (Figure S1). GG-RED performed equally well, with 94.74% correct “B” diagnostic reads and 99.35% correct “A” diagnostic reads (Figure S1). DNOVO also performed well with 90.65% correct “B” and 99.45% correct “A” diagnostic reads (Figure S1).

### Developmental gene networks

Self-Organizing Maps was used to identify developmental gene networks. Because 22 different combinations of tissue/ontogeny were investigated, gene networks were identified in three different groups: vegetative, including leaves, roots, and vegetative shoot tip; reproductive, including reproductive shoot tip, flowers, and geocarpic development up until Pattee stage 3; and seed development starting at Pattee stage 5 through stage 10.

### Vegetative networks

**Figure 2:**
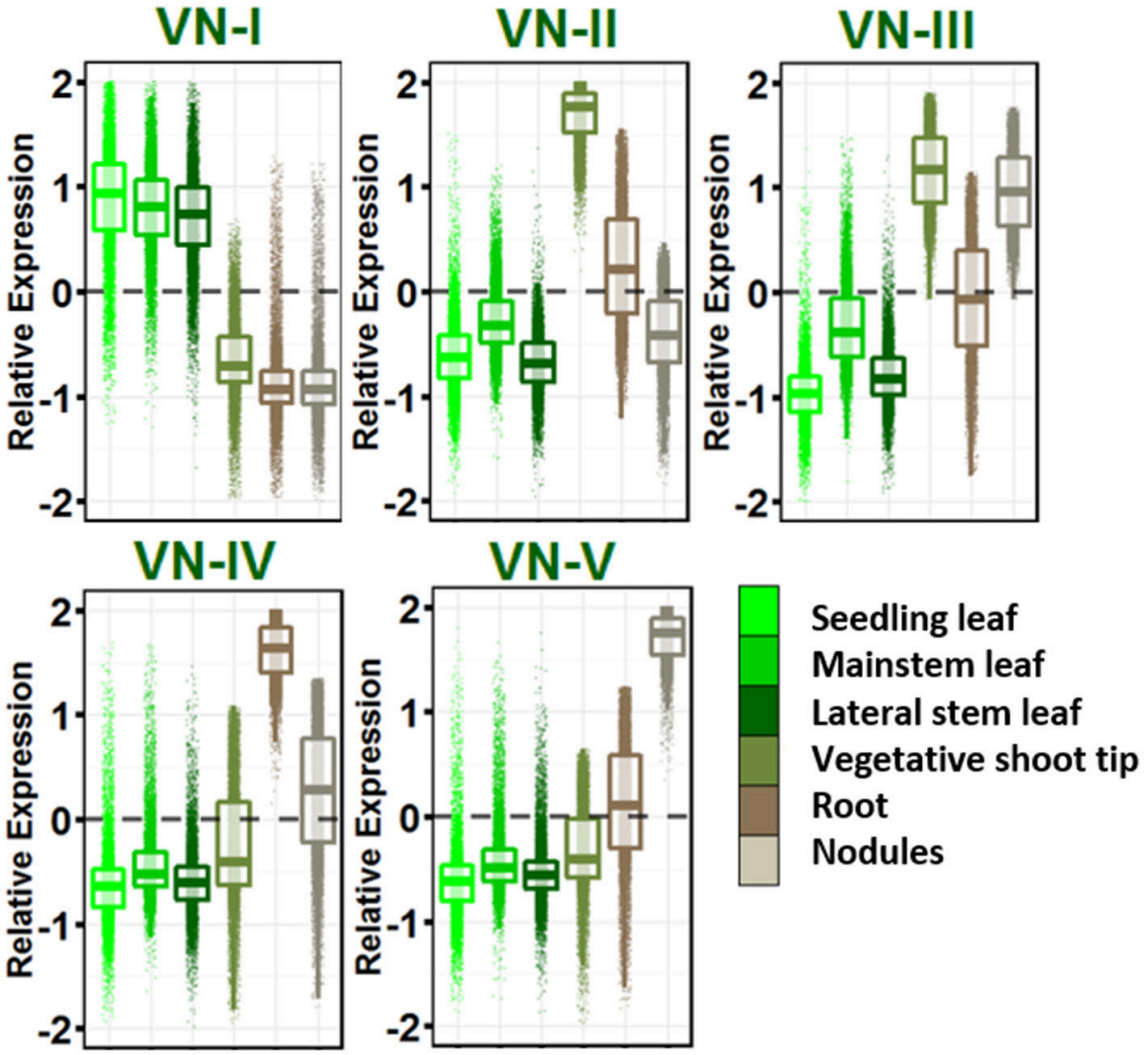
**Vegetative gene networks**. Relative expression is Z-score transformed FPKM. Plots are jigger boxplots with every gene's normalized gene expression shown in the plot. Boxplot is traditional boxplot with median, and upper and lower quartiles. Each tissue is colored as in key to right in the same order.

Five dominant gene expression patterns were revealed in vegetative tissues (Figure 2) and genes in each group with their gene ontologies are listed in Table S8. VN-I showed increased expression in leaves of all ontogenies sampled relative to other vegetative tissues. This network is enriched for pathways known to be active in leaves: photosynthesis, sugar metabolism and starch biosynthesis, auxin and brassinosteroid biosynthesis, and other secondary metabolism including carotenoids, flavonoids, terpenes, and cutin.

VN-II showed increased expression in vegetative shoot tip relative to other vegetative tissues. This network is enriched with mitosis-related, auxin signaling and polar efflux, meristem maintenance and regulation, and organ morphogenesis genes.

VN-III included genes enriched for higher expression in vegetative shoot tips and root nodules. This network is highly enriched for transcription/translation and the spliceosomal complex GO terms.

VN-IV showed root enriched expression and VN-V showed nodule enriched expression. These networks provide a contrast between root growth and nodulation/nitrogen fixation. Network IV is enriched for ubiquitin ligase activity, response to stress and defense response, signal transduction, hyper sensitive response and systemic acquired resistance, lignin biosynthesis, and cytokinin biosynthesis.

VN-V, in contrast, is enriched for glycolysis and tricarboxylic acid cycle, trehalose biosynthesis and trehalose phosphatase activity, resveratrol biosynthesis, ethylene biosynthesis and signaling, and amino acid biosynthesis. Both Networks IV and V are enriched for nodulation.

### Reproductive networks

**Figure 3:**
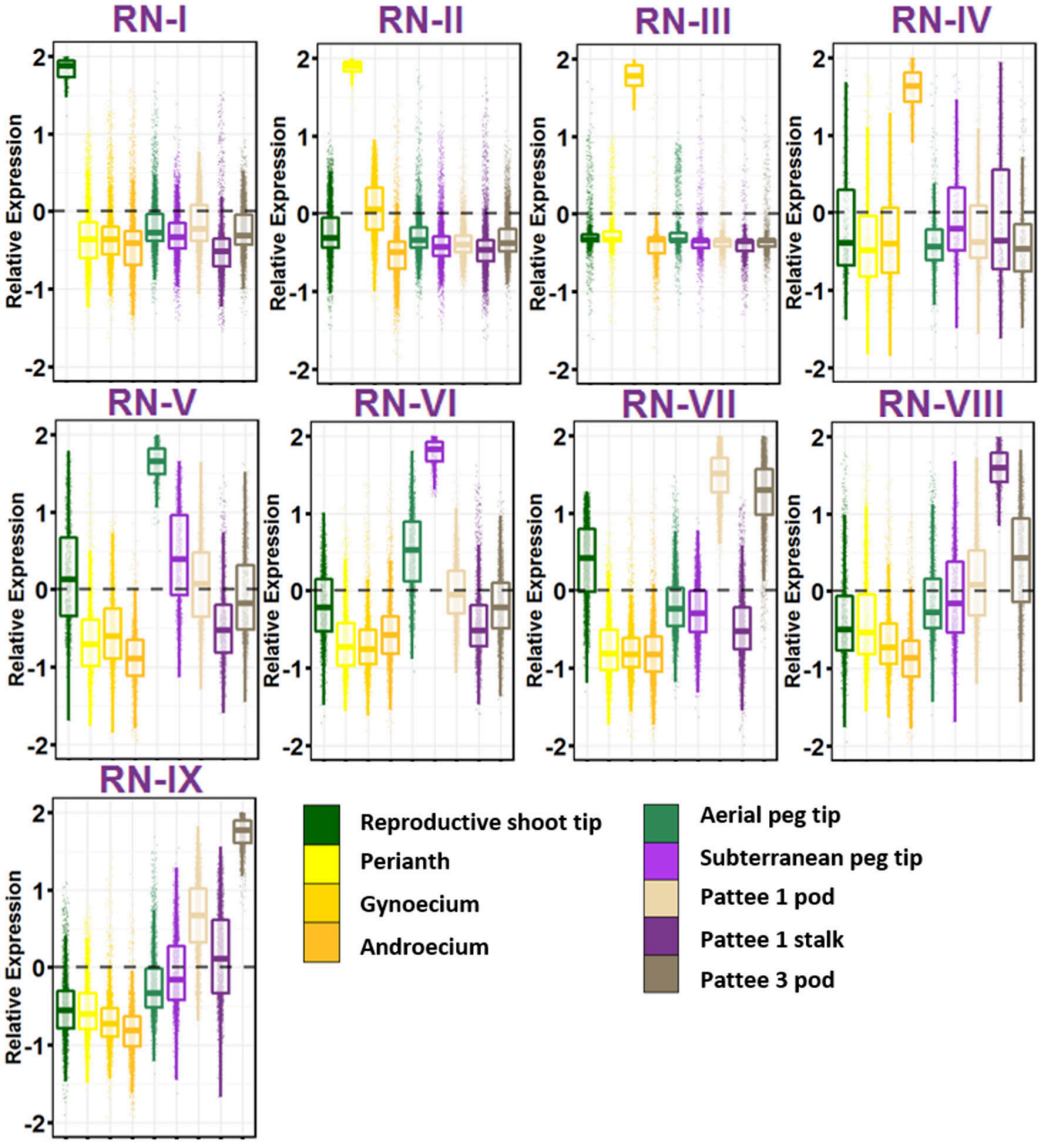
**Reproductive Gene Networks (RN)**. Relative expression is Z-score transformed FPKM. Pattee refers to the stages of peanut development established in Pattee et al. (1974). Plots are jigger boxplots with every gene's normalized gene expression shown in the plot. Boxplot is traditional boxplot with median, and upper and lower quartiles. Each tissue is colored as in key to right in the same order.

The group of geocarpic development-related samples revealed a fascinating set of nine gene networks that show increased expression stepwise in one of the nine ontogenies sampled (Figure 3; Table S9). RN-I, with enriched expression in reproductive shoot tips was enriched for pathways involved in flower development including transcription factor activity, specification of floral organ identity, specification of organ position, and pollen formation. Also enriched in RN-I include genes involved in vegetative to reproductive phase transition of meristem, the control of which differentiates the flowering pattern between subspecies *hypogaea* and *fastigiata*. The one hormone pathway enriched in this cluster is brassinosteroid biosynthesis and homeostasis.

RN-II, with high expression in perianth i.e., flower petals, keel, and hypanthium tissue is enriched for auxin activated signaling. In contrast to RN-I, RN-II is enriched for transporter activity, specifically glucose, galactose, GDP-mannose, folic acid, D-xylose, glycerol, and myo-inositol transmembrane transporter activity. RN-II is also enriched for hormone signaling and biosynthesis including auxin, cytokinin, brassinosteroid, and jasmonic acid.

RN-III shows high expression in gynoecium, and is enriched for fertilization and pollen tube growth, showing that even as flowers were collected at 8:30 a.m. some flowers were already fertilized. RN-III also has top enriched GO terms microtubule, microtubule motor activity, and associated complex, cell wall, pectate lyase and pectinesterase activity, and protein autophosphorylation.

RN-IV includes genes with high expression in androecium. This network is enriched for anther development, RNA binding, mitochondrial, electron transport, ATP synthesis, and ubiquinone activity.

The first ontogeny of geocarpic development sampled, the tip of elongated aerial pegs, is represented in RN-V. RN-V is enriched for photosynthesis and chlorophyll synthesis, an observation made previously in elongating pegs (Zhu et al., 2014). Additionally the network is enriched for flavonoid and oxylipin biosynthesis, lipid catabolism, and cytokinin signaling.

When the peg reaches the soil, the tip begins to swell into the pod. The mechanism for this is thought to be mechano- and light-sensitive. RN-VI represents genes highly expressed in the peg tip after soil penetration for 24 h. This network is enriched for response to stress, specifically heat stress and light. This has also previously been observed in gene expression studies (Zhu et al., 2014). Further, RN-VI is enriched for cell wall and secondary cell wall biogenesis, photorespiration, copper ion binding, protein folding, and translation.

RN-VII represents genes highly expressed in the early stages of pod development; pod expansion after soil penetration (Pattee 1). One major difference between RN-V and VI, and RN-VII is that RN-VII is highly enriched for transcription factor activity (adjusted *p* = 1.00E-08). After elongation and penetration, expansion of the developing pod is initiated by major transcriptional reprogramming, supported by these gene networks. In addition, RN-VII is enriched for cotyledon and leaf development, response to cadmium ion, ethylene signal activation, positive gravitropism, and root hair development. This set of genes is also elevated in expression in RN-IX (Pattee 3 pod) suggesting that these genes maintained the high expression level in young pod development.

Genes highly expressed in the stalk of the elongated peg are represented in RN-VIII. The stalk is interesting in that it has the anatomical structure of a stem, with vascular bundles forming a ring around a pith in the center, but it behaves like a root with its positive gravitropism. RN-VIII is enriched for photosynthesis. Hormone pathways enriched in RN-VIII are ethylene-activated signaling and response to cytokinin. Also enriched is response to water deprivation.

Finally, grouped with the reproductive stages, RN-IX represents genes highly expressed in the pod at Pattee stage 3. This network is in contrast to RN-VII in that genes in Network IX are expressed at a lower level in Pattee stage 1 pods and represent elevated expression in the later stage after initial pod swelling.

### Seed development networks

**Figure 4:**
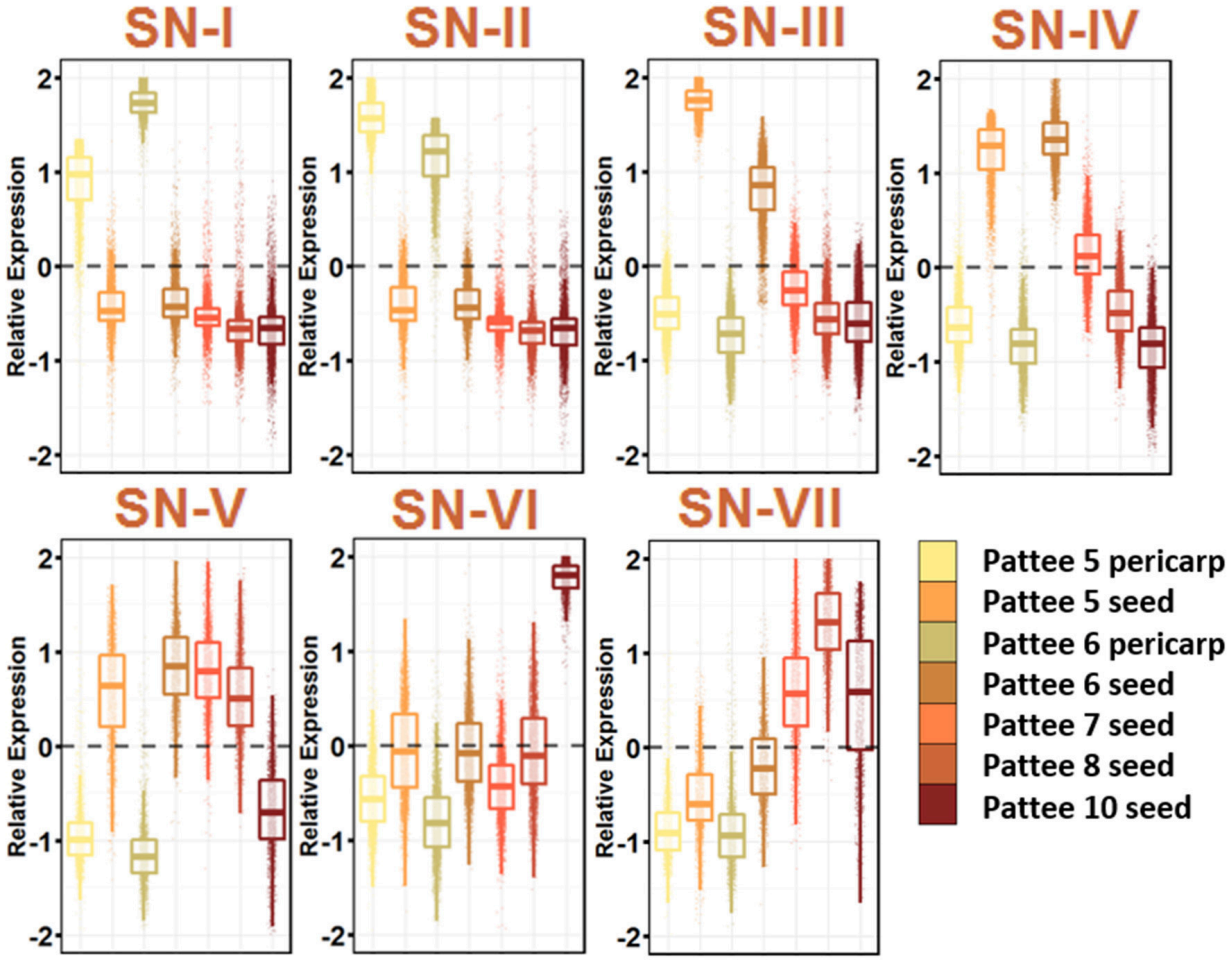
**Seed Development Gene Networks (SN)**. Relative expression is Z-score transformed FPKM. Pattee refers to the stages of peanut development established in (Pattee et al., 1974). Plots are jigger boxplots with every gene's normalized gene expression shown in the plot. Boxplot is traditional boxplot with median, and upper and lower quartiles. Each tissue is colored as in key to right in the same order.

Seed expansion began at Pattee stage 5, where sugar content reached a maximum in the pericarp and the seed began differentiating from the pericarp (Pattee et al., 1974). The seed development networks (SN) represent 7 networks that describe the seed development, including pericarp-specific expression (Figure 4; Table S10).

SN-I represents genes highly expressed in pericarp tissue at Pattee stage 5 and 6. Stage 6 is where the inner pericarp tissue begins to show cracks (Pattee et al., 1974). SN-I is enriched for response to chitin, bacterium and fungus exposure, secondary cell wall biogenesis, cellulose biosynthesis, ethylene activated signaling, and response to water deprivation. Transcriptional changes continue to occur in the pericarp tissue as SN-I is enriched for transcription factor activity.

SN-III, with genes showing high expression in Pattee stage 5 seeds and slightly lower expression in stage 6 seeds, is highly enriched for cell division. The earlier seed ontogeny sampled was Pattee stage 3, and it is clear that between stage 4 and 5, the developing seed is undergoing rapid cell division. SN-IV also represents genes highly expressed in seed stage 5 and 6, with stage 6 having slightly higher expression and then stage 7 intermediate expression. This SN represents an expression program slightly delayed from SN-III.

At Pattee stage 5 and continuing into stage 6, a group of genes is highly expressed (SN-IV) and then decreases in expression as seed development progresses. Top enriched genes in this network include photosynthesis, photosystem I and II, chloroplast, chlorophyll binding, chlorophyll biosynthesis, mitosis, DNA methylation, microtubule-based movement, glycolysis, and fatty acid biosynthesis. Seeds in other legumes have shown photosynthetic activity during the early stages of development (Borisjuk et al., 2005), and this photosynthetic oxygen evolution is key for accumulation of lipids (Rolletschek et al., 2005). As the seed matures, chloroplasts differentiate into storage plastids (Borisjuk et al., 2005). Peanuts mature underground and do not receive light, yet plastids are essential for fatty acid biosynthesis, so this gene expression during early seed development may be retained even though fruit undergo geocarpic development.

SN-V represents genes expressed at all stages of seed development and decreasing expression at Pattee stage 10. This group represents the constitutively expressed seed development genes. SN-V top enriched GO terms mirror SN-IV with the addition of vegetative phase change, leaf morphogenesis, and transcription.

A group of genes representing increasing expression as seed development proceeds, SN-VII, includes top enriched GO terms nutrient reservoir activity and response to salt stress. These genes represent the accumulation of storage proteins in the developing seed and the desiccation process that occurs as the seed matures.

It is clear from these seed development networks that photosynthesis is an active part of peanut seed development, even showing gene enrichment for circadian rhythm and response to blue light (SN-VII). This remains an interesting aspect of peanut seed development.

### Homeolog expression bias

Homeolog-specific assembly provides the unique position to estimate genome-wide homeolog expression bias in a developmental and tissue-specific context. First, a set of putative homeologous pairs was identified by using best hit reciprocal BLAST of cDNA sequence and predicted ORF protein coding sequence (Table S2). Pairs that had best hits in both directions for both comparisons were considered putative homeologous pairs. Mapping these pairs to the *A. duranensis* and *A. ipaensis* reference genomes and plotting those mapping coordinates against each gene's pair reveals rearrangements shown with comparative mapping of the genomic data (Bertioli et al., 2016; Figure S2). These two lines of evidence provide support for this set of putative pairs. This set comprised 8816 pairs from GG-RED. Expression bias was minimal across all tissues (Table 2; Table S11); only 27 gene pairs showed constitutive bias (13 toward A copy, 14 toward B copy). Of those 27, no pairs were completely dominant in all tissues where one subgenome copy was expressed and the other silenced. Genes showing constitutive “B” genome bias include chloroplastic phosphoglycerate kinase (PGK), an ubiquitin activating enzyme, an ubiquitin hydrolase, a microtubule severing katanin p60 subunit, and glucose-6-phosphate isomerase. Genes showing “A” genome bias include the kinases WNK4, serine/threonine protein kinase, and pyruvate kinase, the microtubule-interacting protein SPIRAL1, EXECUTER1, and a neutral ceramidase.

**Table 2:** **Genome-wide subgenome expression bias**.

	**A-biased**	**B-biased**	**A completely dominant**	**B completely dominant**	**Balanced**
Constitutive	13 (0.15%)	14 (0.16%)	0	0	8789 (99.7%)
Seedling leaf	352 (4.4%)	335 (4.2%)	0	0	7234 (91.3%)
Mainstem leaf	155 (1.9%)	153 (1.9%)	31 (0.38%)	30 (0.37%)	7962 (96.3%)
Lateral leaf	361 (4.4%)	341 (4.2%)	49 (0.6%)	51 (0.62%)	6964 (94.2%)
Vegetative shoot tip	660 (7.9%)	606 (7.3%)	38 (0.46%)	39 (0.47%)	7473 (83.8%)
Root	742 (8.7%)	745 (8.7%)	43 (0.5%)	45 (0.53%)	6990 (81.6%)
Nodules	574 (6.8%)	516 (6.1%)	50 (0.59%)	50 (0.59%)	7288 (86%)
Reproductive shoot tip	781 (9.1%)	790 (9.2%)	43 (0.49%)	38 (0.43%)	6913 (80.7%)
Perianth	922 (11%)	934 (11.19%)	41 (0.49%)	49 (0.59%)	6412 (76.8%)
Gynoecium	110 (1.2%)	130 (1.5%)	20 (0.23%)	20 (0.23%)	8349 (96.8%)
Androecium	59 (0.71%)	58 (0.7%)	16 (0.19%)	21 (0.25%)	8161 (98.1%)
Aerial gynophore tip	184 (2.2%)	219 (2.6%)	27 (0.32%)	30 (0.35%)	8064 (94.6%)
Subterranean gynophore tip	585 (7%)	587 (7.1%)	42 (0.5%)	34 (0.41%)	7076 (85%)
Pattee 1 pod	87 (1%)	66 (0.79%)	20 (0.23%)	24 (0.29%)	8193 (96.7%)
Pattee 1 stalk	129 (1.6%)	107 (1.3%)	29 (0.35%)	33 (0.4%)	8023 (96.4%)
Pattee 3 pod	431 (5%)	409 (4.8%)	31 (0.36%)	36 (0.42%)	7633 (89.4%)
Pattee 5 pericarp	257 (3%)	231 (2.7%)	26 (0.31%)	30 (0.35%)	7966 (93.6%)
Pattee 5 seed	506 (5.74%)	492 (5.58%)	42 (0.48%)	40 (0.45%)	7736 (87.75%)
Pattee 6 pericarp	254 (3%)	246 (2.9%)	19 (0.23%)	33 (0.39%)	7876 (93.5%)
Pattee 6 seed	343 (4%)	342 (4%)	32 (0.38%)	34 (0.4%)	7757 (91.2%)
Pattee 7 seed	273 (3.3%)	241 (2.9%)	36 (0.43%)	24 (0.29%)	7811 (93.2%)
Pattee 8 seed	308 (3.8%)	290 (3.6%)	39 (0.48%)	28 (0.34%)	7478 (91.8%)
Pattee 10 seed	281 (3.6%)	267 (3.4%)	56 (0.71%)	32 (0.41%)	7239 (91.9%)

*For each tissue expression bias between the set of 8816 putative homeologous pairs was calculated using DESeq2. Constitutive refers to the A or B copy and is significantly higher expressed in all tissues sampled. A biased are A pairs significantly higher expressed in A and B biased are B pairs significantly higher expressed in B. Completely A or B dominant are pairs that are expressed in A or B while the other is not expressed. Balanced is no expression difference between the A or B copy. Percentages in parentheses indicate percentage of the total pairs expressed in that tissue type*.

Table 2 shows expression bias for each tissue type and ontogeny assayed in this study. In individual tissues, expression bias was also minimal, with balanced expression between subgenome copies exhibited by 76.8 to 98.1% of expressed pairs. The highest amount of expression bias was seen in perianth only including wings, banner, hypanthium, keel and lower lip of the calyx with 23% of expressed pairs showing bias. The lowest amount of expression bias was seen in androecium with only 1.8% of expressed pairs showing bias. Overall, expression bias was highest in tissues with meristematic activity, including reproductive (20%) and vegetative (16%) shoot tips, roots (19%), nodules (14%), and subterranean peg tip (15%). Expression bias in leaves and developing seeds ranged from 12 to 5% and was lowest in androecium (1.8%) and gynoecium (3%). Within tissue expression bias was also very balanced with only slight differences between number of pairs biased toward the “A” or “B” genome copy. Complete dominance in expression of an “A” or “B” genome copy was also low, with the highest in roots, nodules, and perianth at 1% of expressed pairs.

**Figure 5:**
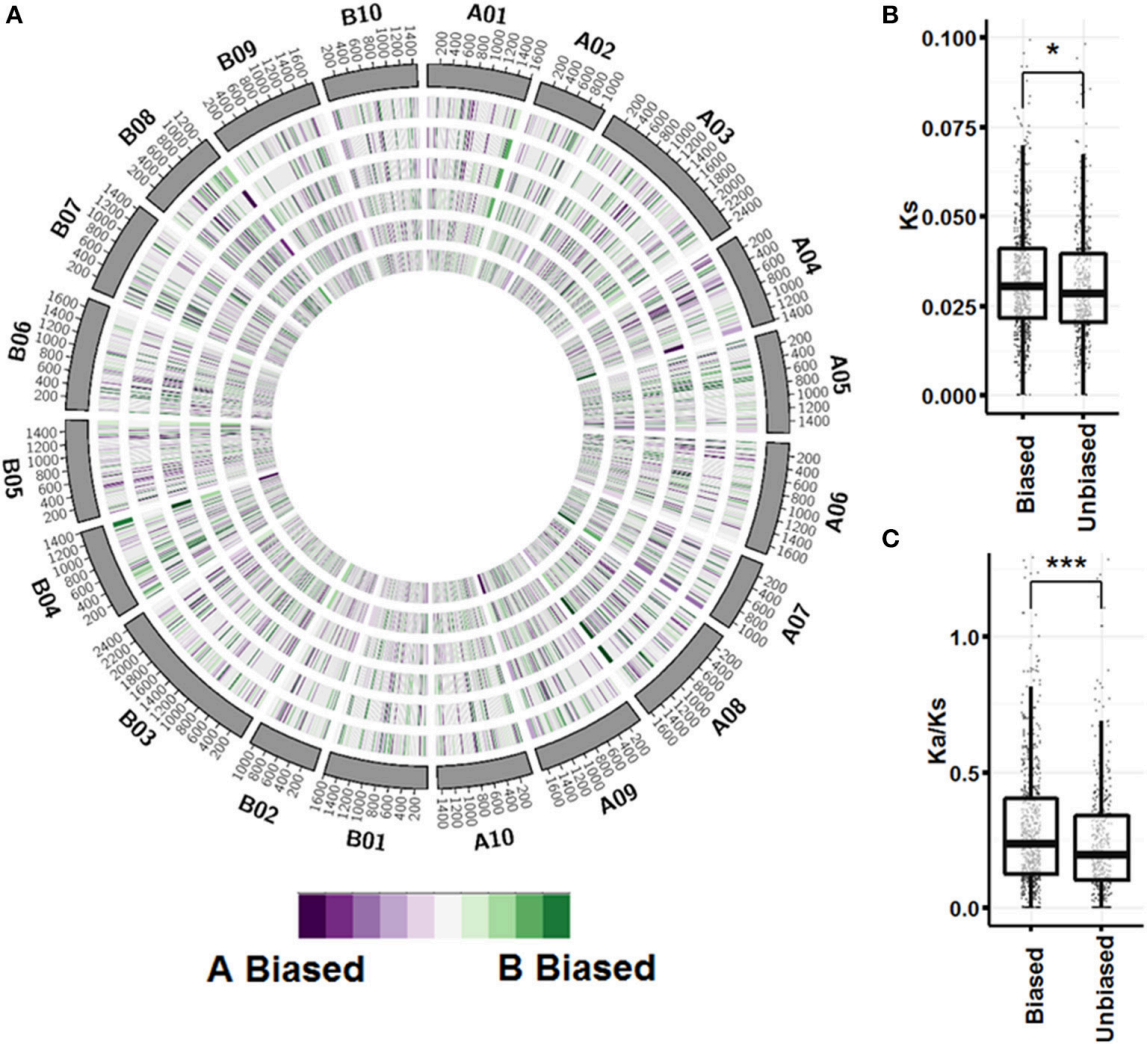
**Subgenome expression bias. (A)** Circos plot showing expression bias by gene pair position for vegetative tissues. Homeologous pairs were mapped to *A. duranensis* and *A. ipaensis* pseudomolecules and assigned a position on the mapped chromosome. Pairs that did not map to their reciprocal chromosomes were not considered. A sliding window of 50 loci in 10 loci increments was used to visualize bias. From outer ring to inner ring; seedling leaf, mainstem leaf, lateral leaf, vegetative shoot tip, roots, nodules. **(B)** Synonymous substitution rate between unbiased pairs and pairs with significant expression bias in at least 3 tissues (p < 0.05 by Wilcox on rank test). **(C)** Non-synonymous/Synonymous substitution rate between unbiased and biased pairs (*p* < 0.0001 by Wilcox on rank test).

A global view of expression bias reveals that although most genomic regions are regulated the same across tissue types, there are regions that are oppositely regulated between tissue types and ontogenies (Figure 5; Figure S3). Further inspection, however, shows that individual homeologous pairs do not use alternate subgenome bias between tissues, only showing balanced expression or expression bias in one subgenome. Synonymous substitution rate (K_s_) between pairs showing expression bias in at least three tissues (*n* = 749) is slightly greater than pairs showing no expression bias in any tissue (*n* = 441) (*p* < 0.05; Figure 5B). Between biased pairs and pairs showing no bias, the rate of non-synonymous substitution/synonymous substitution (Ka/Ks) is larger, suggesting that the rate of evolution after tetraploidization is a driving factor controlling subgenome expression bias (Figure 5C).

### Alternative splicing

The transcriptional landscape of alternative splicing in the context of tissue- and ontogeny-specific usage was investigated. Using Finesplice (Gatto et al., 2014), 204,503 splice junctions were identified across all tissues and ontogenies. The splice junctions were mined for alternative splicing events using custom scripts (Table S3) and further characterized into exon skipping, alternative 5′ donor, and alternative 3′ acceptor. A total of 9026 alternative splicing (AS) events were identified. Of those, 1010 were exon skipping events, 4293 were alternative 5′ donor events, and 3723 alternative 3′ acceptor events (Table S3). Of the alternative 3′ acceptor events, 287 NAGNAG events were further discovered (Figure S4). NAGNAG events consist of the alternative splicing of 3 bases within the motif NAGNAG. The splicing event occurs proximal and splices off one NAG or distal and retains the entire motif.

**Figure 6:**
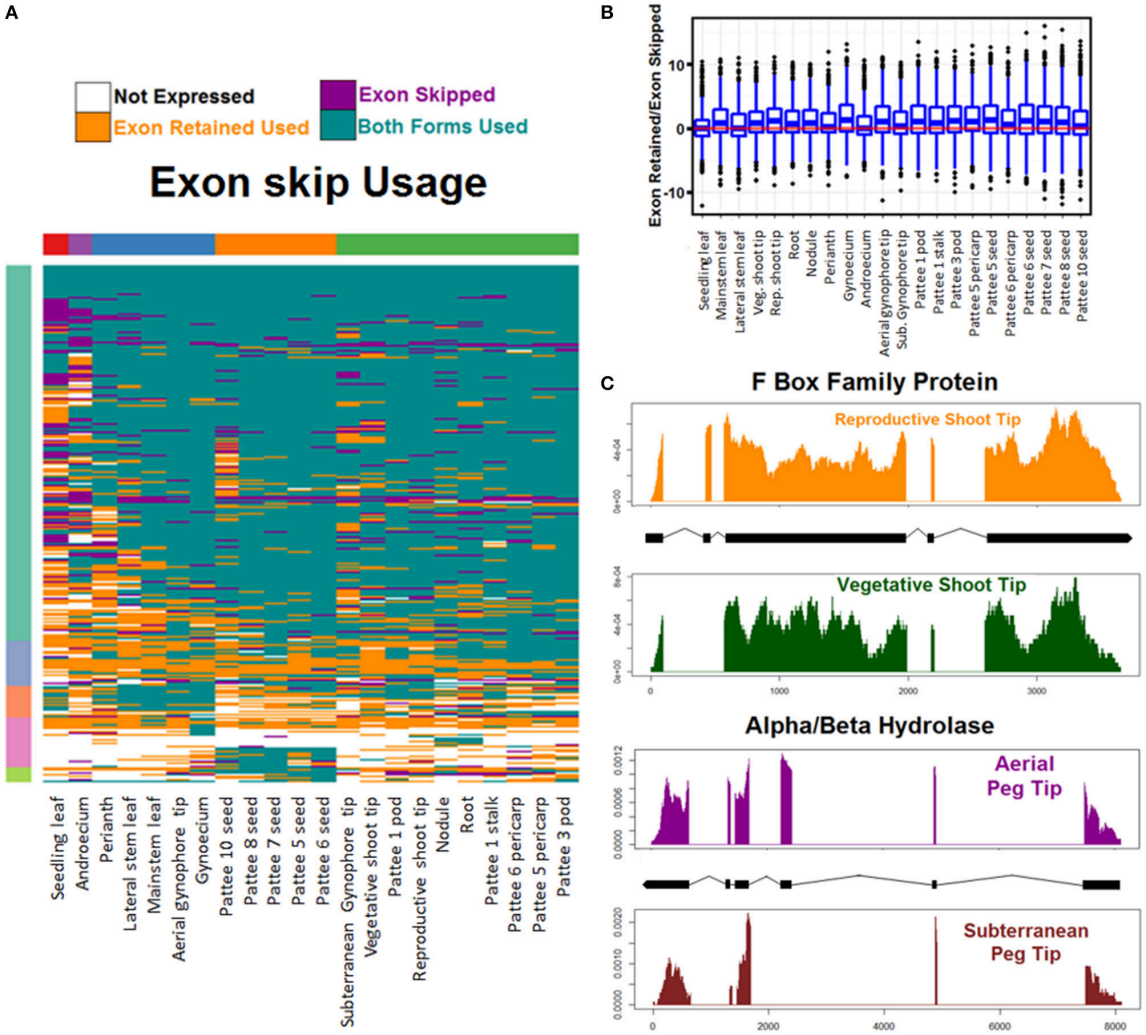
**Exon skipping alternative splicing (AS). (A)** Hierarchical clustering of usage of exon skipping AS events. Tissues (columns) and events (rows) are clustered for Euclidean distance of exon skipping AS event usage. **(B)** Exon skipping usage preference. For each tissue and all exon skipping AS events the preference to either skip the exon or retain it is calculated as log2 (Reads showing exon retain/Reads showing exon skip). Balanced usage (0) is indicated with a red line. **(C)** Two examples of exon skipping AS preferential usage. Read coverage represented as read density of mapped reads/total reads mapped to transcript. X axis is base pairs of transcript.

Usage was calculated across all tissue types of each AS event and was determined to be both events if any replicate showed evidence of both splicing events. Overall, both events were used in the majority of cases (Figure 6A, Figure S5). Both exon skipping events were used in 65% of cases, both alternative 5′ donor events were used in 66% of cases, and both alternative 3′ acceptor events were used in 70% of cases. Hierarchical clustering reveals that AS usage clusters in a predictable manner by tissue type with seedling leaf exhibiting the most unique AS usage pattern. Of the other tissues, seeds cluster together and subterranean peg tip clusters with peg stalk, early developing pods, and pericarp. Interestingly, aerial peg tip clusters with reproductive shoot tip (Figure 6A, Figure S5).

Additionally, the preference of each tissue toward one AS event or its alternative was examined (Figure 6B and Figure S6). Finesplice determines how many reads support a particular splice junction. For each AS event, the log base 2 transformed fold change of the reads supporting one event over the alternative was calculated. Globally, for alternative acceptor and donor AS events, there is no bias toward the proximal or distal version (Figure S6). For exon skipping AS events however, there is preference globally to retain the exon over skipping it (Figure 6B).

This set of AS events is the first global description of alternative splicing events in *A. hypogaea*. It represents a rich resource to explore the genetic regulation of peanut development. Figure 6C shows two interesting examples of exon skipping events with alternative forms in similar tissues undergoing differential development. The top example shows the read coverage of an F Box family protein that is differentially spliced in vegetative shoot tips and reproductive shoot tips. Exon two is skipped in reproductive shoot tips and is retained in vegetative shoot tips. The bottom example shows an alpha/beta hydrolase that retains exon three in actively dividing peg tip above ground, but skips the exon when it is below ground. These two examples and others like them may provide insight into the regulation of key events in peanut development.

### Long non-coding RNAs

Eliminating redundancy from the GG assembly reduced the assembly size from 183,062 to 80,326 transcripts. These eliminated transcripts, however, show dynamic expression patterns. These transcripts were mined for possible long non-coding RNAs. After first selecting 77,937 transcripts that had no predicted ORF, had no significant hit to known proteins, and had no annotated Pfam domain from the Trinotate annotation, all transcripts that had an average expression across all 58 libraries of less than 2 FPKM were filtered out. Additionally, after applying the Coding Potential Assessment Tool (CPAT) (http://lilab.research.bcm.edu/cpat/, Wang et al., 2013), only 289 additional transcripts were determined to be coding. This filtering method left 6407 putative long non-coding RNAs (lncRNAs) (Tables S5–S7).

**Figure 7:**
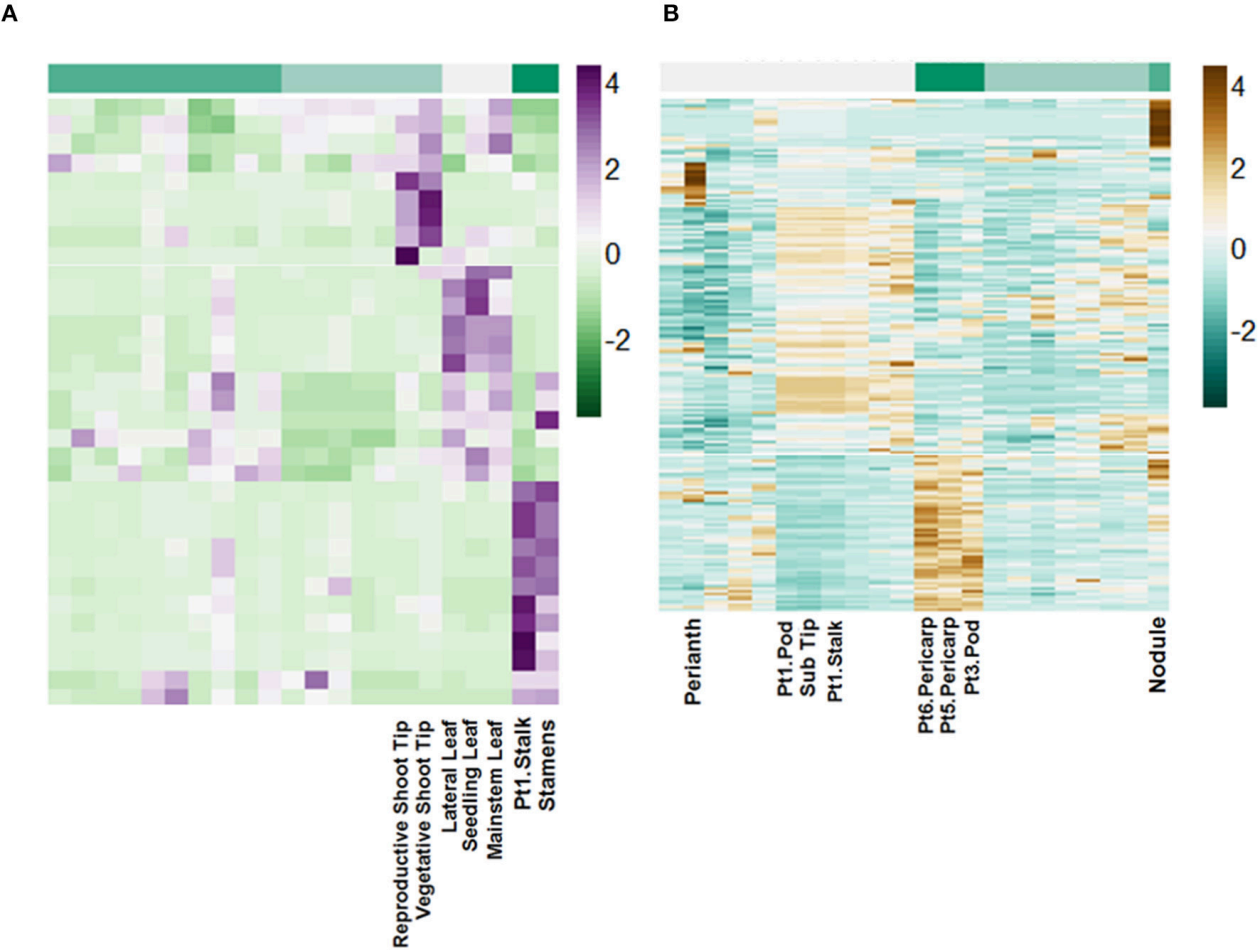
**Tissue-specific regulation of putative lncRNAs**. Scale is Z-score normalized relative expression. **(A)** Regulation of selected pri-miRNA transcripts. **(B)** Regulation of selected intergenic lncRNA transcripts.

Using miRBase 21 (http://www.mirbase.org/) 72 pri-miRNA transcripts with matches by length greater than 94% and greater than 80% nucleotide identity were identified (Table S4). These putative pri-miRNAs are dynamically regulated and in some cases highly expressed. For example, a transcript matching peu-MIR2916 (*Populus euphratica*) has peak expression of above 6000 FPKM. Additionally, clustering of the expression patterns of these transcripts reveals they are highly regulated in a tissue-specific manner (Figure 7).

The remaining putative lncRNAs were further characterized as intergenic, intronic, and exonic using the *A. duranensis* and *A. ipaensis* gene annotations. Intergenic lncRNAs were found to be more than 200 bp away from annotated genes. Intronic lncRNAs overlapped with a predicted mRNA but did not overlap with an exon, whereas exonic lncRNAs overlapped with at least one predicted exon either in the sense or antisense direction. We identified 2195 intergenic lncRNAs (Table S5), 3858 exonic lncRNAs (Table S6), and 189 intronic lncRNAs (Table S7). Clustering of the expression profiles of these lncRNAs reveals tissue-specific expression patterns (Figure 7; Figure S7). These expression patterns provide evidence that these putative lncRNAs may have roles during peanut development. This resource will be important for the study of peanut genetic regulation and is the first global set of lncRNAs for *Arachis*.

## Discussion

A well-documented consequence of allopolyploidization, the phenomenon of two genomes merging and doubling, homeolog expression bias has been shown in many allopolyploids (Bottley et al., 2006; Hovav et al., 2008; Akhunova et al., 2010; Koh et al., 2010; Zhou et al., 2011). A homeolog-specific transcriptome assembly for peanut allowed investigation of genome-wide expression bias. Homeolog expression bias of 8816 putative homeolog pairs was similar to that seen in other polyploids; most homeolog pairs showed balanced expression. In a tissue-specific context, expression bias ranged from 1.8 to 23.2% of pairs. In cotton, mid-parental levels of expression was reported for 70 to 81% of tested gene pairs (Flagel et al., 2008; Yoo et al., 2013). In hexaploid wheat, 81% of pairs were shown to be expressed at mid-parent levels (Akhunova et al., 2010). Overall, expression bias was balanced between the A and B genomes of peanut, with a slight bias toward A genome copies. Nevertheless, there were striking tissue-specific differences in expression bias with vegetative and reproductive shoot tips and perianth showing the highest number of pairs with expression bias. The gene pairs with the strongest bias tended to be biased in most of the tissues sampled with tissue-specific differences being between pairs showing more subtle expression differences. These differences could represent false positives or indicate that tissue-specific expression bias is driven by smaller differences in expression.

We identified 9026 alternative splicing (AS) events. There has been extensive work investigating the extent and role of alternative splicing in plants with roles in all aspects of growth and development, stress response, and defense response (Filichkin et al., 2010, 2015; Mastrangelo et al., 2012; Wang et al., 2012; Staiger and Brown, 2013; Capovilla et al., 2015). Here, we have not investigated stress response and so only profiled developmentally-related AS. There are interesting developmental transitions in peanut, including the transition from gravitropic growth of the gynophore to a subterranean swelling pod, and the determination of a vegetative shoot or reproductive shoot. Our analysis has identified 12 differential exon usage, 528 differential 5′ donor sites, and 441 differential 3′ acceptor sites between genes expressed in vegetative and reproductive shoot tips. This differential usage of isoforms of the same gene describe the difference between vegetative determination and reproductive determination as much as differential gene expression. In Arabidopsis, flowering time is controlled by the splicing of *FCA* (Quesada et al., 2003), and this control is conserved in rice (Lee et al., 2005). Although no evidence of the AS in orthologs of *FCA* was found in peanut, the differential AS usage in these two organ fates may play a role in the transition between vegetative and reproductive shoots.

The expression patterns of putative non-coding RNAs suggest tissue- and ontogenic-specific regulation. A classic gene regulating nodulation, *GmENOD40*, is a non-coding RNA (Yang et al., 1993). No ortholog of *GmENOD40* was found in our transcriptome assembly or in the diploid genome assemblies (Bertioli et al., 2016), although a group of intergenic lncRNAs was specifically expressed in nodules. This tissue-specific expression suggests these lncRNAs play a role in regulating nodulation in peanut. Similarly, a group of putative pri-miRNA transcripts was identified and displayed enriched expression in the vegetative and reproductive shoot tips, including sequences that match mir319 and mir166, micro RNAs that have been shown to regulate cell proliferation and shoot apical meristem development respectively (Zhang and Zhang, 2012; Schommer et al., 2014). Up-regulation of miRNA binding and miRNA metabolic process was also identified in these tissues in the gene network analysis.

Our identified co-expression networks represent a valuable new resource for researchers to find target candidate genes regulating traits of interest. Geocarpy is a fascinating aspect of *Arachis* reproductive development. There have been studies investigating the gene expression during geocarpic development in peanut (Xia et al., 2013; Zhu et al., 2014; Chen et al., 2016). Zhu et al. (2014) and Xia et al. (2013) identified an up-regulation of photosynthesis in aerial pegs. RN-V, genes up-regulated in aerial peg tips, confirms this observation with enrichment of chloroplast and response to light. Interestingly, no genes annotated as regulating gravitropism or response to gravitropism in RN-V were identified. It has been suggested that the gravitropic response in peanut gynophores is regulated by the localization of starch grains by gravity, the starch statolith hypothesis (Moctezuma and Feldman, 1999). Although our data cannot confirm this, there is a conspicuous lack of enriched expression for gravitropism in the aerial peg tip. However, two genes (both homeologs of each) involved in positive gravitropism in RN-VI which contains genes up-regulated in subterranean peg tips were identified. These two genes are putative orthologs of *PINOID* and *TAA1*, both implicated in regulation of auxin-regulated gravitropism (Sukumar et al., 2009; He et al., 2011). After soil penetration, auxin localization shifts to the peg tip which undergoes increased growth in terms of cell number and volume (Moctezuma, 1999). This increased localization of auxin is possibly the cause of the horizontal growth that occurs after soil penetration and allows the peg tip to burrow underneath the soil (Moctezuma, 1999). The enriched expression of *TAA1* and *PINOID* in subterranean peg tips suggest that the increased auxin localization is due to localized auxin biosynthesis via the IPA pathway, of which *TAA1* is the first step, and localization to the tip by *PINOID*-regulated auxin transport. This observation shows the strength of the gene atlas for candidate gene selection to test hypotheses as two strong candidate genes have been identified for further investigation that may play a key role in geocarpic development.

The four botanical types of peanut are split into two subspecies, *hypogaea* and *fastigiata*, in which a main difference between them is that *fastigiata* produces reproductive nodes on the main stem whereas *hypogaea* does not. Our gene atlas contains vegetative shoot tips and reproductive shoot tips which are represented by the co-expression networks VN-II and RN-I, respectively. Investigating these co-expression networks may provide candidate genes for the change from a vegetative to a reproductive meristem. RN-I is enriched for the GO term “vegetative to reproductive phase transition of meristem.” Of those genes, 18 are not represented in VN-II and are potential candidate genes. One gene, a putative ortholog of *LUMINIDEPENDENS* (*LD*), a floral transition promoter is an interesting candidate (Lee et al., 1994; Aukerman et al., 1999). Perhaps a more intriguing candidate is *HEADING DATE 3* (*Hd3a*), which is a putative ortholog of *FT* in Arabidopsis, *Hd3a* in rice, and *SFT* in tomato (Lifschitz et al., 2006; Tamaki et al., 2007). *Hd3a* has been shown to encode a mobile floral-promoting signal in rice, a short-day flowering plant (Tamaki et al., 2007). Peanut, on the other hand, is a day neutral plant. *SFT* was shown to induce flowering in day neutral tomato and tobacco (Lifschitz et al., 2006). The putative ortholog in peanut is also highly expressed in lateral leaves, but not expressed at all in main stem leaves. Tifrunner belongs to subspecies *hypogaea*. It will be interesting to investigate the expression of this gene in main stem leaves of subspecies *fastigiata* genotypes. There are other flower promoting candidate genes that are expressed highly in reproductive shoot tips, but are also highly expressed in vegetative shoot tips on the main stem. The gene atlas helped identify the difference between the two meristems to hypothesize candidate genes for the regulation of transition to the reproductive meristem in peanut that may differentiate the two subspecies of *A. hypogaea*.

**Figure 8:**
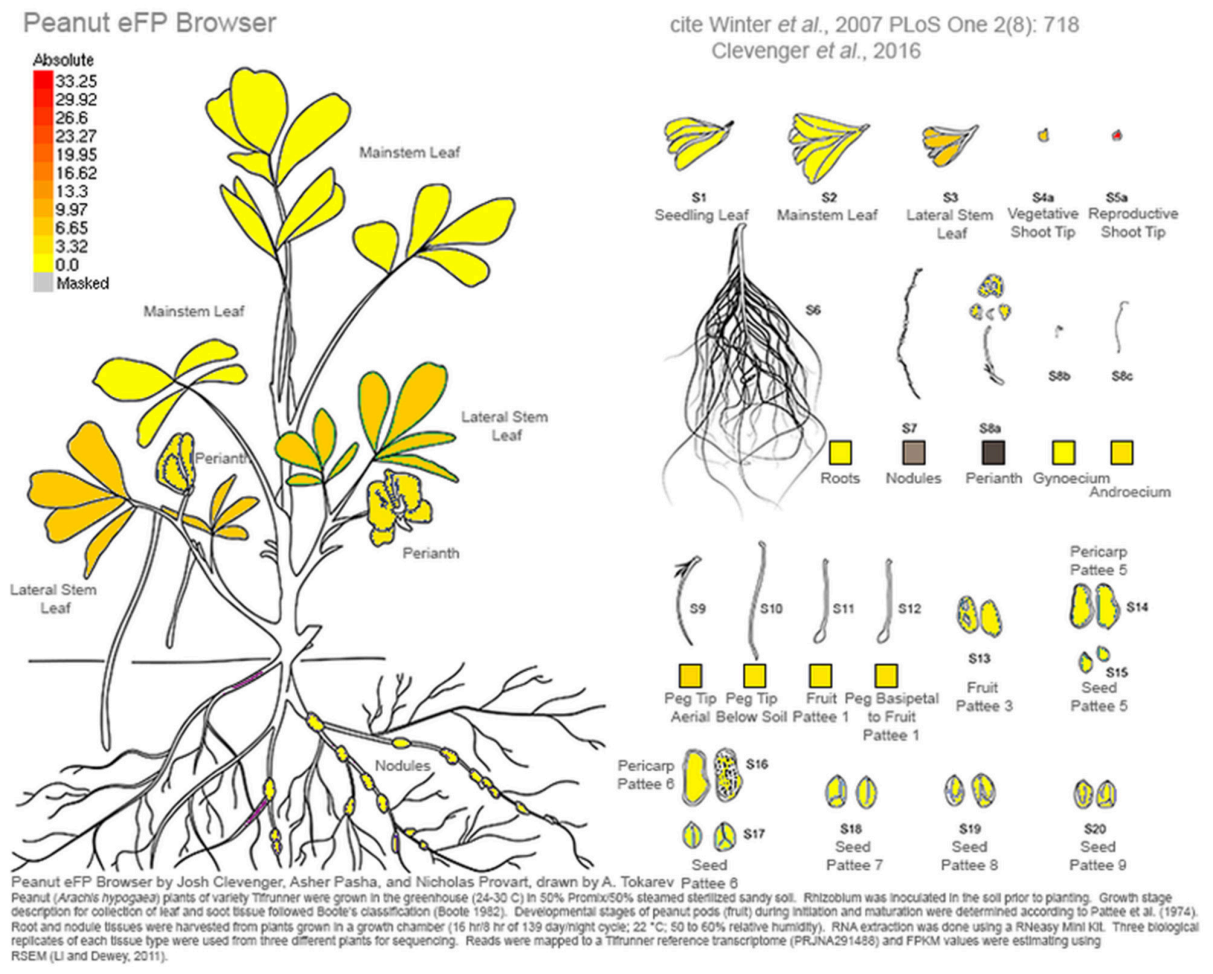
**Screen shot from the ***Arachis*** eFP browser showing expression for the putative ortholog of ***Hd3a*** and it's enriched expression in reproductive shoot tips**. Expression is in FPKM.

This work represents a major new resource for peanut genomics. As an allotetraploid of very recent origin, the unraveling of homeologous gene copies in a homeolog-specific assembly allows the resolution needed to investigate the genetics of cultivated peanut. A comprehensive gene atlas has been constructed using 22 tissue types and ontogenies spanning the development of peanut. These data are available currently at peanutbase.org as expression tracks and incorporated into an efp browser (Winter et al., 2007) for *Arachis* (http://bar.utoronto.ca/efp_arachis/cgi-bin/efpWeb.cgi; Figure 8). There are efp browsers for many other crops and model species including Arabidopsis (Winter et al., 2007), tomato (http://bar.utoronto.ca/efp_tomato/cgi-bin/efpWeb.cgi), maize (http://bar.utoronto.ca/efp_maize/cgi-bin/efpWeb.cgi), soybean (http://bar.utoronto.ca/efpsoybean/cgi-bin/efpWeb.cgi), rice (http://bar.utoronto.ca/efprice/cgi-bin/efpWeb.cgi), and others. These browsers are powerful tools for all aspects of genomics. Additionally, all identified putative alternative splicing events and non-coding RNAs will also be incorporated into peanutbase.org for peanut researchers to access. Ultimately this work represents a paradigm shift in peanut from using high- throughput sequencing to generate resources to using it to define and test hypotheses. This shift will mark an increase in the efficiency of discovery using genomics in *Arachis*.

## Author contributions

PO, BS, and YC conceptualized the research; YC and BS provided genetic resources and data; JC and YC performed experiments, conducted data analysis, and curated data; JC and PO contributed to methodology; JC wrote the original draft and was responsible for data visualization; PO, YC, BS, and JC revised the manuscript; PO administered the project.

### Conflict of interest statement

The authors declare that the research was conducted in the absence of any commercial or financial relationships that could be construed as a potential conflict of interest.
