# Supplementary material for: A Developmental Transcriptome Map for Allotetraploid Arachis hypogaea
Source: Front Plant Sci. 2016 Sep 30;7:1446. doi: 10.3389/fpls.2016.01446 (PMC5043296; doi:10.3389/fpls.2016.01446)
Supplement: Supplementary file 12 [file Image1.PDF]

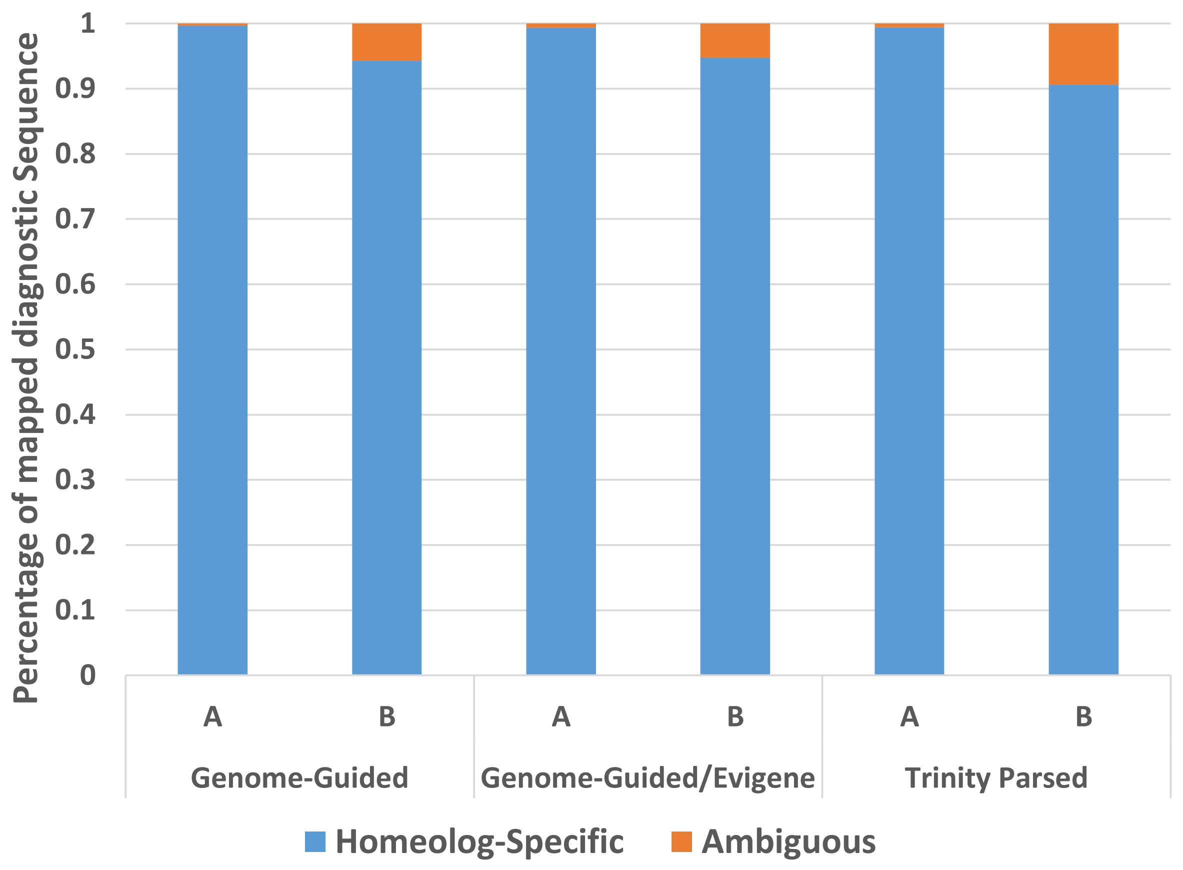

Figure S1: Accuracy of assembled transcripts. Diagnostic 100 bp sequences differing between A and B genome by 1 mismatch were mapped to the transcript assemblies. A genome diagnostic sequence mapping to A-derived transcripts, as well as B genome diagnostic mapping to B-derived transcripts were counted as homeolog-specific. Ambiguous sequences were described as diagnostic sequences mapping to the opposite genome-derived transcript.
