# Supplementary material for: A Developmental Transcriptome Map for Allotetraploid Arachis hypogaea
Source: Front Plant Sci. 2016 Sep 30;7:1446. doi: 10.3389/fpls.2016.01446 (PMC5043296; doi:10.3389/fpls.2016.01446)
Supplement: Supplementary file 13 [file Image2.PDF]

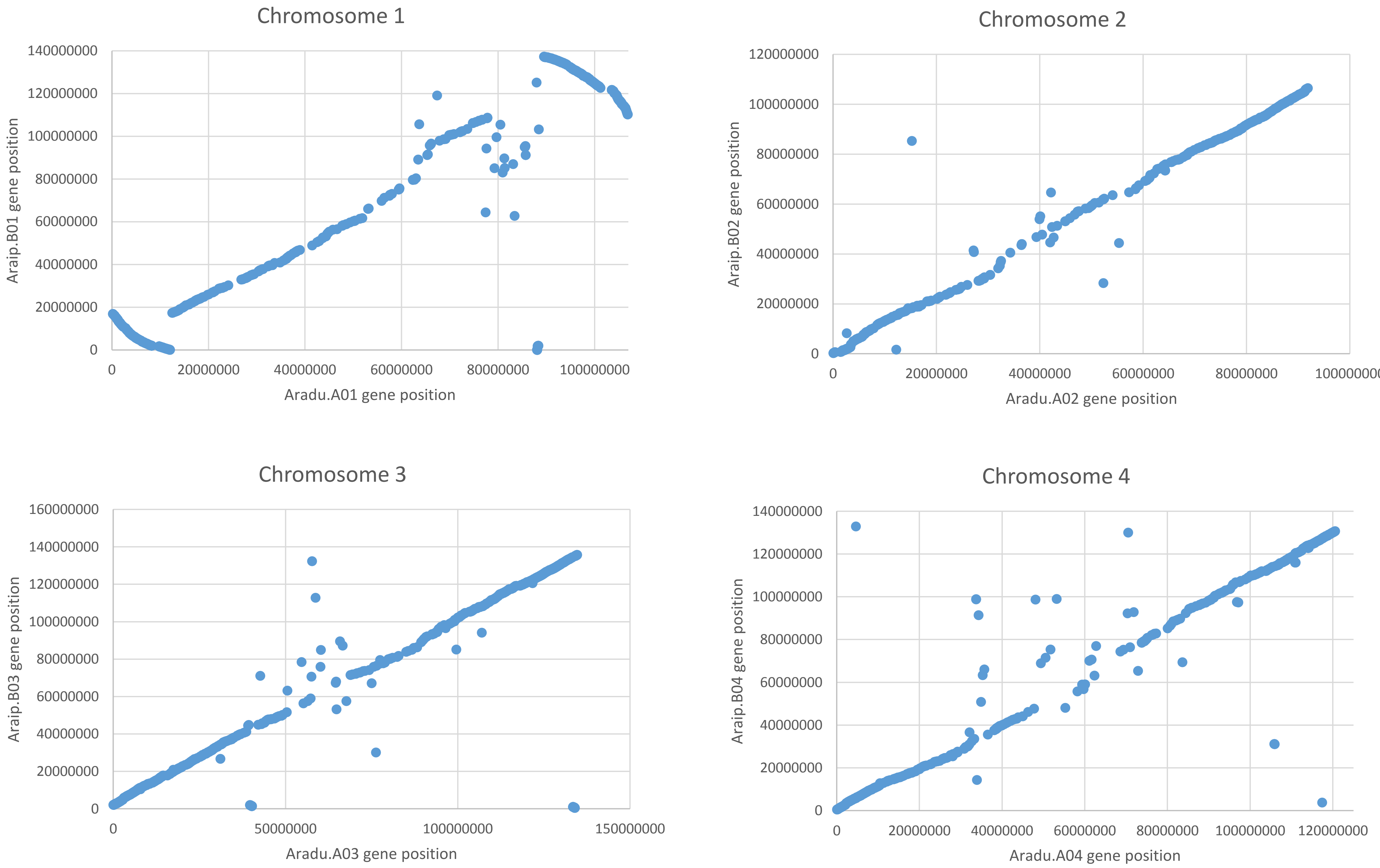
Chromosome 1
Araip.B01 gene position
Aradu.A01 gene position
0
20000000
40000000
60000000
80000000
100000000
120000000
140000000
Chromosome 2
Araip.B02 gene position
Aradu.A02 gene position
0
20000000
40000000
60000000
80000000
100000000
120000000
Chromosome 3
Araip.B03 gene position
Aradu.A03 gene position
0
20000000
40000000
60000000
80000000
100000000
120000000
140000000
160000000
50000000
150000000
Chromosome 4
Araip.B04 gene position
Aradu.A04 gene position
0
20000000
40000000
60000000
80000000
100000000
120000000
140000000

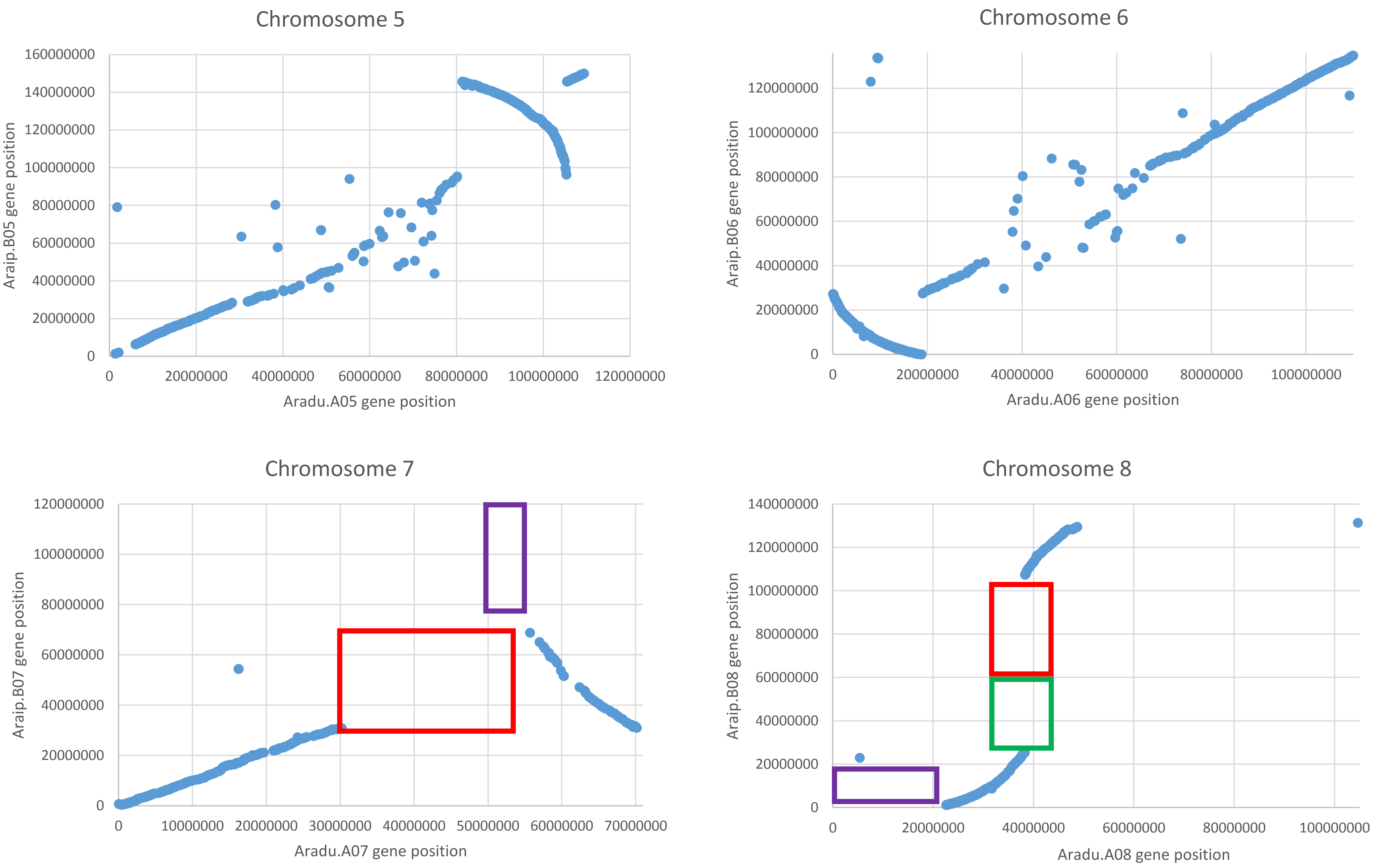

Chromosome 5
Araip.B05 gene position
Aradu.A05 gene position
0
20000000
40000000
60000000
80000000
100000000
120000000
140000000
160000000
Chromosome 6
Araip.B06 gene position
Aradu.A06 gene position
Chromosome 7
Araip.B07 gene position
Aradu.A07 gene position
10000000
30000000
50000000
70000000
Chromosome 8
Araip.B08 gene position
Aradu.A08 gene position

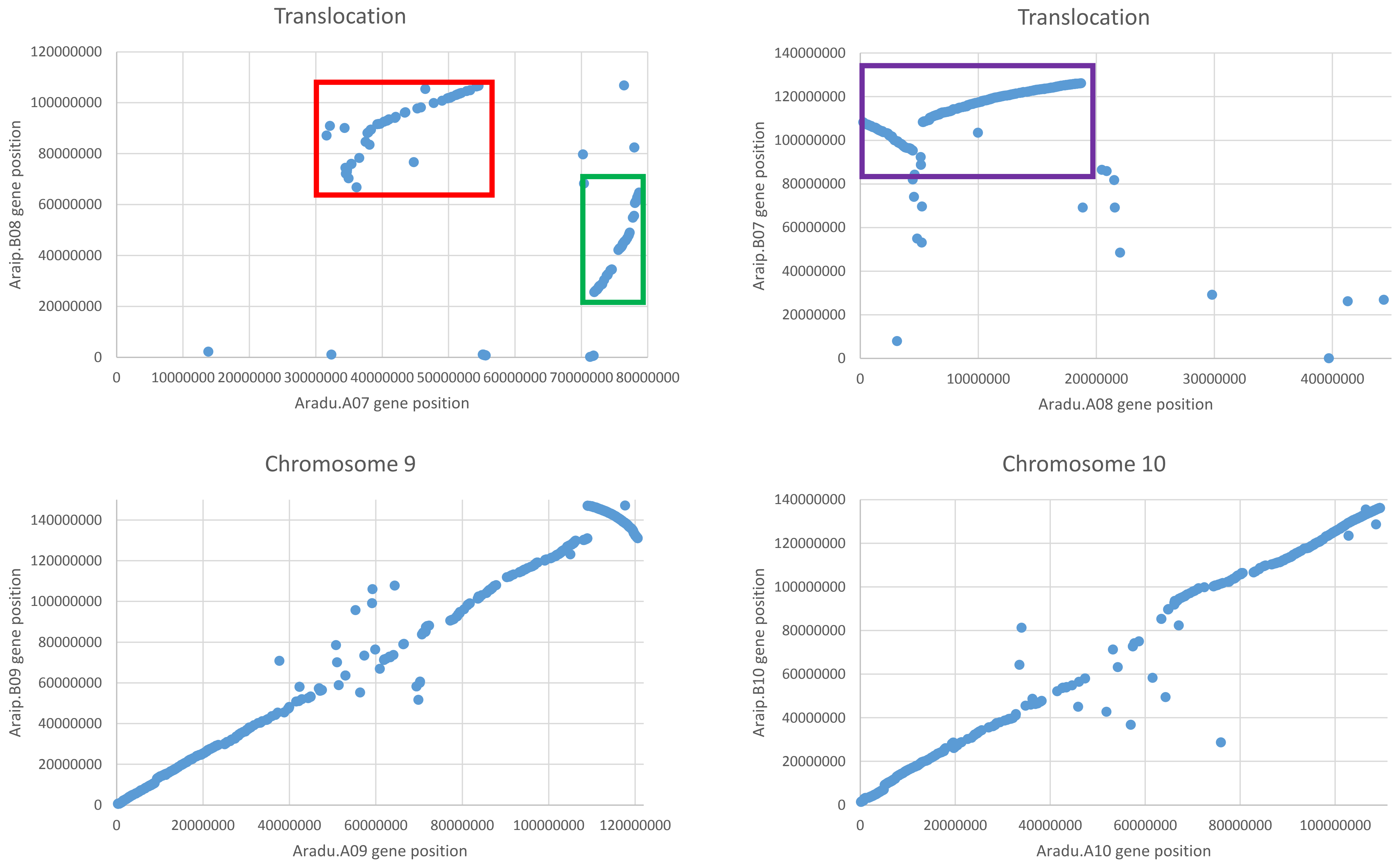
Translocation
Araip.B08 gene position
0
20000000
40000000
60000000
80000000
100000000
120000000
Aradu.A07 gene position
0
10000000
20000000
30000000
40000000
50000000
60000000
70000000
80000000
Translocation
Araip.B07 gene position
0
20000000
40000000
60000000
80000000
100000000
120000000
140000000
Aradu.A08 gene position
0
10000000
20000000
30000000
40000000
Chromosome 9
Araip.B09 gene position
0
20000000
40000000
60000000
80000000
100000000
120000000
140000000
Aradu.A09 gene position
0
20000000
40000000
60000000
80000000
100000000
120000000
Chromosome 10
Araip.B10 gene position
0
20000000
40000000
60000000
80000000
100000000
120000000
140000000
Aradu.A10 gene position
0
20000000
40000000
60000000
80000000
100000000
