# Supplementary material for: A Developmental Transcriptome Map for Allotetraploid Arachis hypogaea
Source: Front Plant Sci. 2016 Sep 30;7:1446. doi: 10.3389/fpls.2016.01446 (PMC5043296; doi:10.3389/fpls.2016.01446)
Supplement: Supplementary file 14 [file Image3.PDF]

a

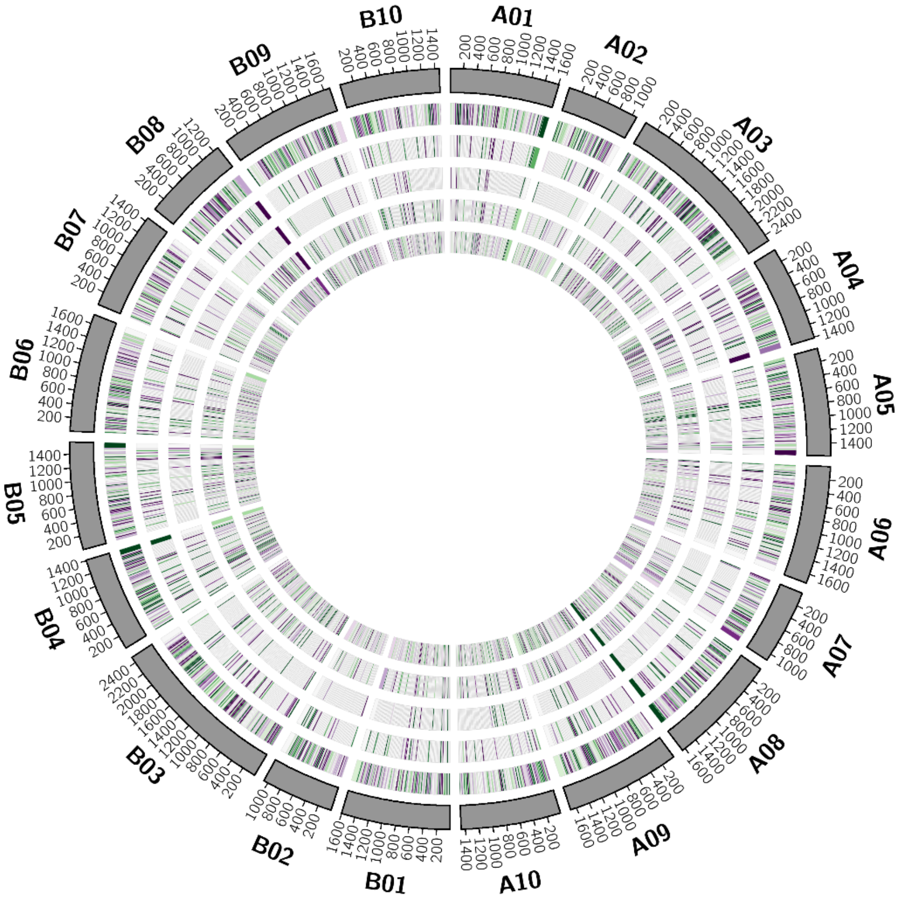

Figure S3: Subgenome expression bias in reproductive organs. Circos plot showing expression bias by gene pair position for reproductive tissues. Homeologous pairs were mapped to *A. duranensis* and *A. ipaensis* pseudomolecules and assigned a position on the mapped chromosome. Pairs that did not map to their reciprocal chromosomes were not considered. A sliding window of 50 loci in 10 loci increments was used to visualize bias. From outer ring to inner ring; Perianth, Pistils, Stamens, Aerial Peg Tip, Subterranean Peg Tip

b

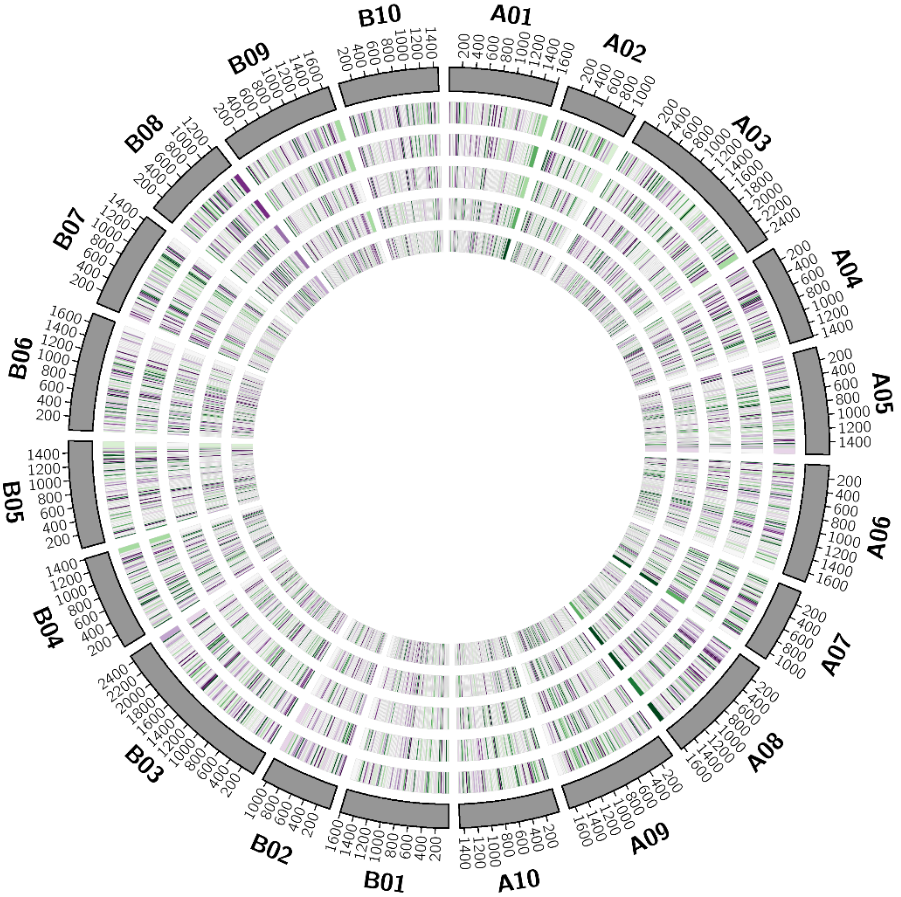

Subgenome expression bias during seed development. Circos plot showing expression bias by gene pair position for reproductive tissues. Homeologous pairs were mapped to *A. duranensis* and *A. ipaensis* pseudomolecules and assigned a position on the mapped chromosome. Pairs that did not map to their reciprocal chromosomes were not considered. A sliding window of 50 loci in 10 loci increments was used to visualize bias. From outer ring to inner ring; Seed Pattee 5, Seed Pattee 6, Seed Pattee 7, Seed Pattee 8, Seed Pattee 10
