# Supplementary figures and images for: A Developmental Transcriptome Map for Allotetraploid Arachis hypogaea
Source: Front Plant Sci. 2016 Sep 30;7:1446. doi: 10.3389/fpls.2016.01446 (PMC5043296; doi:10.3389/fpls.2016.01446)

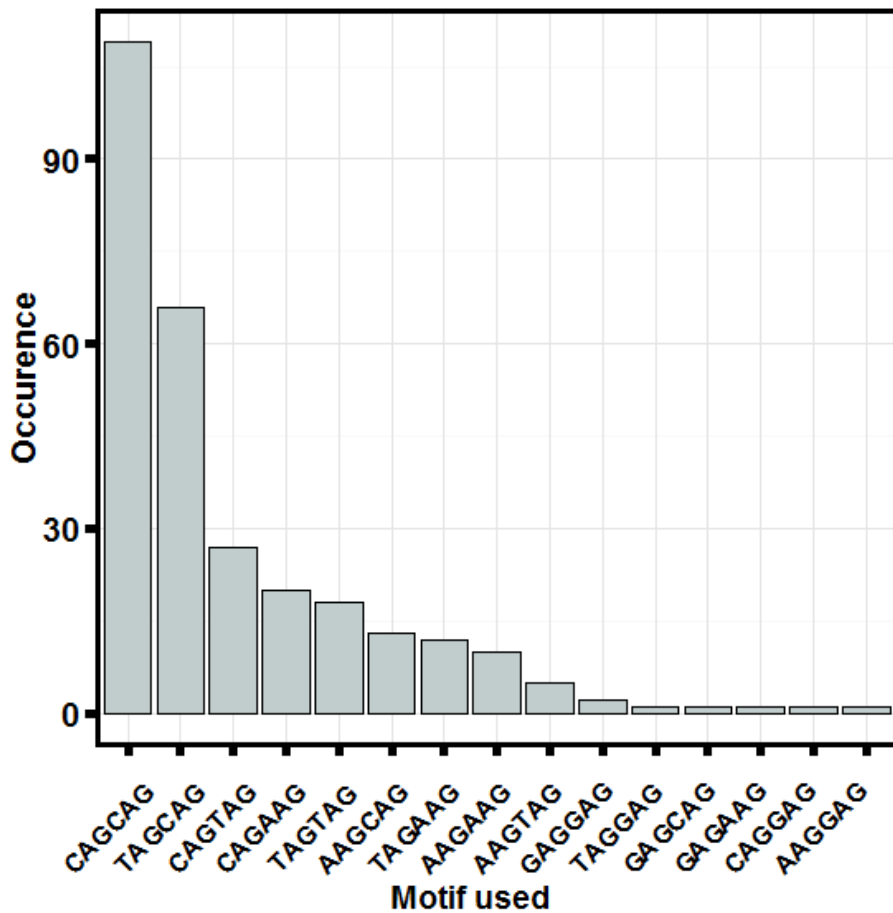

**Figure S4:** Motif usage for NAGNAG AS Alternative 3' Acceptor events

Supplement: Supplementary file 15 [file Image4.PDF]
