# Supplementary material for: A Developmental Transcriptome Map for Allotetraploid Arachis hypogaea
Source: Front Plant Sci. 2016 Sep 30;7:1446. doi: 10.3389/fpls.2016.01446 (PMC5043296; doi:10.3389/fpls.2016.01446)
Supplement: Supplementary file 16 [file Image5.PDF]

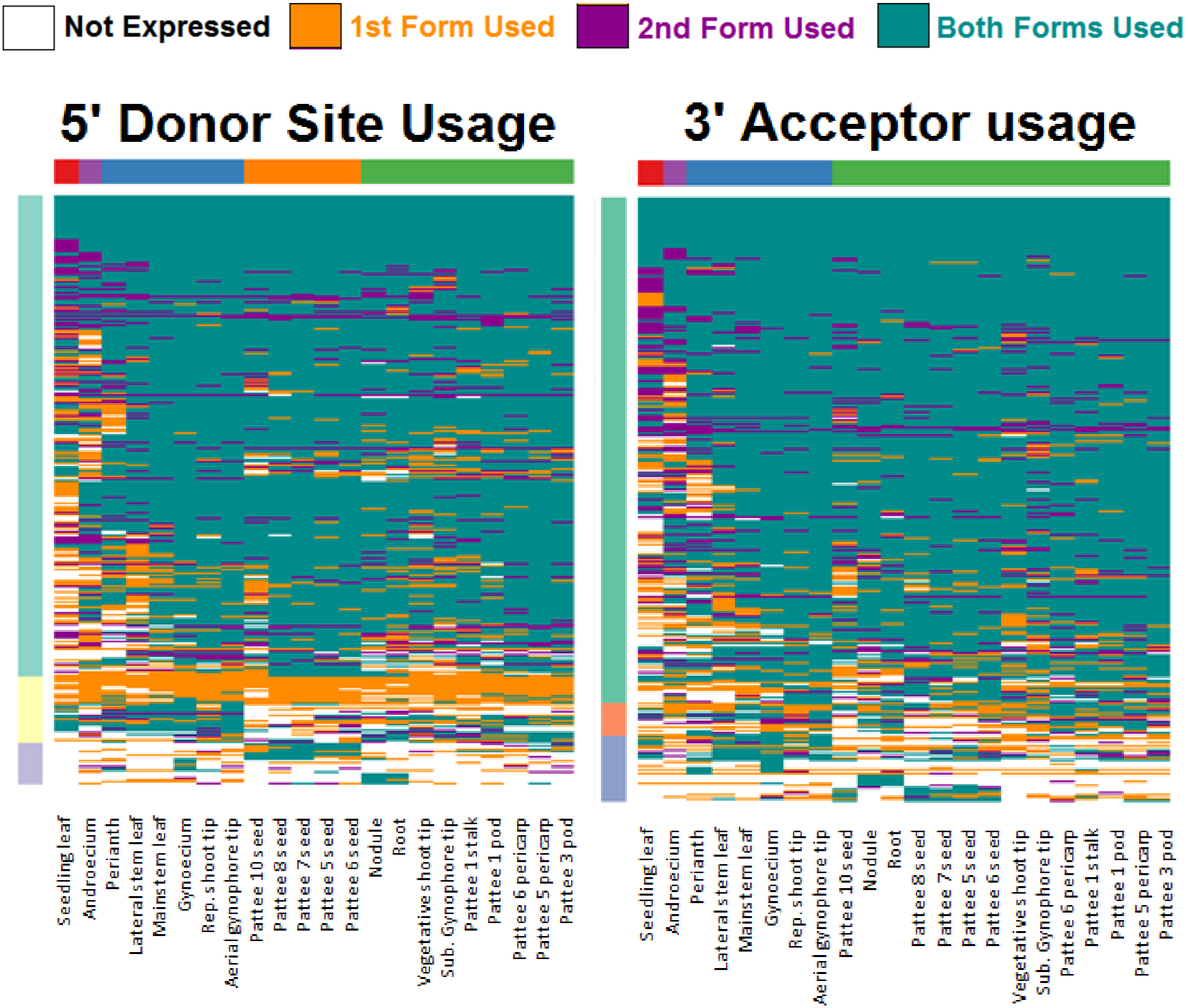

Figure S5. 5' Donor and 3' Acceptor alternative splicing (AS). Hierarchical clustering of usage of AS events. Tissues (columns) and events (rows) are clustered for Euclidean distance of exon skipping AS event usage.
