# Supplementary material for: A Developmental Transcriptome Map for Allotetraploid Arachis hypogaea
Source: Front Plant Sci. 2016 Sep 30;7:1446. doi: 10.3389/fpls.2016.01446 (PMC5043296; doi:10.3389/fpls.2016.01446)
Supplement: Supplementary file 17 [file Image6.PDF]

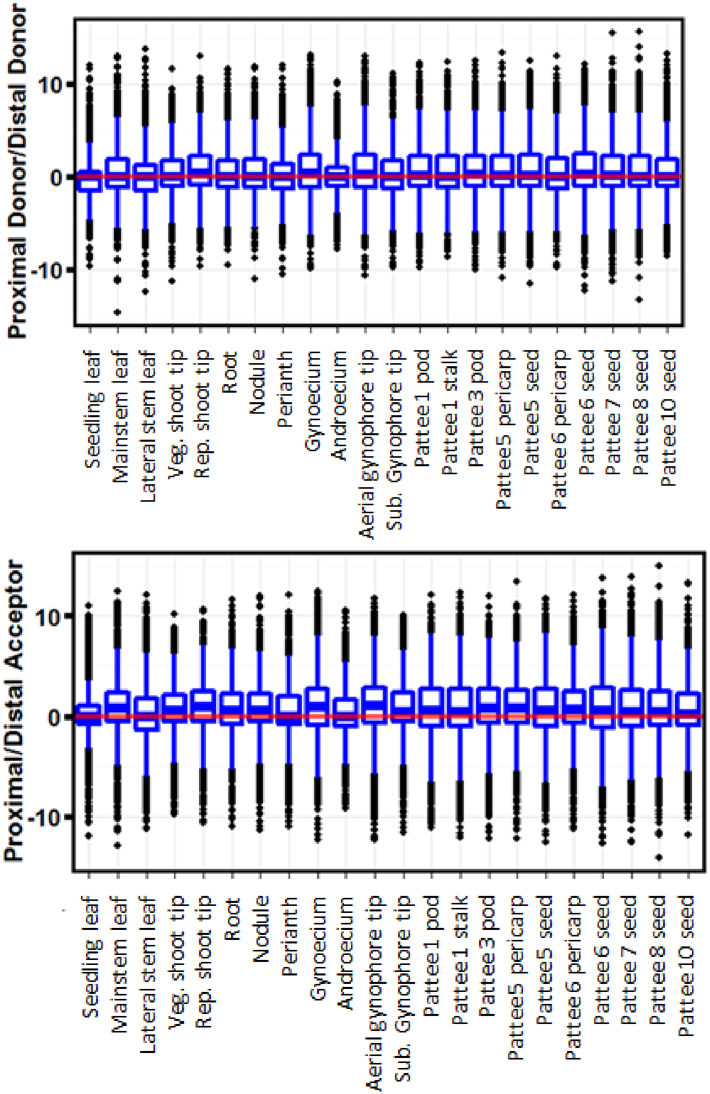

Figure S6. 5’ alternative donor (above) and 3’ alternative acceptor (below) usage preference. For each tissue and all AS events the preference to use the proximal or distal form is calculated as log2 (Reads showing exon retain/Reads showing exon skip). Balanced usage (0) is indicated with a red line.
